# *p*H-selective mutagenesis of protein–protein interfaces: *In silico* design of therapeutic antibodies with prolonged half-life

**DOI:** 10.1002/prot.24230

**Published:** 2012-12-12

**Authors:** Velin Z Spassov, Lisa Yan

**Affiliations:** Accelrys, 10188 Telesis CourtSuite 100, San Diego, California 92121

**Keywords:** binding affinity, mutation, scanning mutagenesis, *p*H, protein ionization, generalized born, CHARMm, antibody, FcRn, neonatal Fc receptor, ImG immunoglobulin G

## Abstract

Understanding the effects of mutation on pH-dependent protein binding affinity is important in protein design, especially in the area of protein therapeutics. We propose a novel method for fast *in silico* mutagenesis of protein–protein complexes to calculate the effect of mutation as a function of pH. The free energy differences between the wild type and mutants are evaluated from a molecular mechanics model, combined with calculations of the equilibria of proton binding. The predicted pH-dependent energy profiles demonstrate excellent agreement with experimentally measured pH-dependency of the effect of mutations on the dissociation constants for the complex of turkey ovomucoid third domain (OMTKY3) and proteinase B. The virtual scanning mutagenesis identifies all hotspots responsible for pH-dependent binding of immunoglobulin G (IgG) to neonatal Fc receptor (FcRn) and the results support the current understanding of the salvage mechanism of the antibody by FcRn based on pH-selective binding. The method can be used to select mutations that change the pH-dependent binding profiles of proteins and guide the time consuming and expensive protein engineering experiments. As an application of this method, we propose a computational strategy to search for mutations that can alter the pH-dependent binding behavior of IgG to FcRn with the aim of improving the half-life of therapeutic antibodies in the target organism.

## INTRODUCTION

Understanding the effects of mutation on protein binding affinity is important in protein design, especially in the area of protein therapeutics. *In silico* predictions of the mutation effects may help guide the experiment and reduce the cost of bringing therapeutics to market. While a number of different methods are available for this purpose,^1^–^4^ most of them do not take into account of the pH-dependency of protein ionization and are applicable only to structures in a predetermined protonation state. Several experimental studies have demonstrated that a change of solution pH within a relatively narrow range could have a significant effect on the binding affinity^5^,^6^ of protein complexes. The pH-dependent binding profile of a protein often plays an important role in the biological function of the protein. For example, immunoglobulin G (IgG) strongly binds to neonatal Fc receptor (FcRn) in endosome at pH 6.0, and dissociates effectively from FcRn in serum at pH 7.4. This pH-selective binding is the key to enable the transport of maternal antibodies to the offspring across the placenta in humans or across the epithelial-cell layers in rodents. Recently, it has been established experimentally, that this pH-dependent binding profile of IgG to FcRn is related to the half-life of IgG in serum.^7^ Engineered monoclonal antibodies (mAbs) with moderately increased binding at both low pH and at pH 7.4 have shown increased serum half-life.^8^ On the other hand, strongly increasing the binding of IgG to FcRn across all pH range does not improve its half-life and the binding of IgG to FcRn at pH 7.4 may even accelerate the clearance of IgG.^7^ This demonstrates the importance of optimizing the pH-dependent binding behavior of protein and calls for theoretical methods which can predict mutation energies at different solution pH. In addition to interaction with FcRn, pH-dependent binding of antibody to its target antigen also has effect on its serum half-life. Most antibodies bind to one target antigen throughout their lifetime due to the target mediated lysosomal degradation. However, a recent example^9^ shows that optimizing the pH-selective binding of an antibody to interleukin-6 receptor (IL-6R) allowed the antibody to be recycled in the host system. An engineered antibody, tocilizumab, which retains the binding to IL-6R in plasma (pH 7.4), but dissociates quickly from IL-6R in acidic endosome, reduces the lysosomal degradation of the antibody and allows the antibody to be recycled back to the plasma and bind to another IL-6R molecule. Another similar example^10^ is the pH selective binding of an engineered antibody to Proprotein Convertase Substilisin Kexin type 9 (PCSK9) which can more effectively reduce the concentration of low density lipoprotein (LDL-cholesterol) in serum. Depending on the purpose of the antibody, its pH-dependent FcRn binding profile can be optimized differently. Most therapeutic antibodies which target specific antigen should be optimized to prolong the half-life so that they can be administered at lower dose and frequency. On the other hand, conjugates of mAb and small molecule inhibitors used to target specific cancer cells could be engineered to have reduced affinity to FcRn in endosome so the drug molecules can be released in cancer cells more efficiently.

All of the recent experimental advances and limitations motivated us to develop a new method which can predict the pH-dependent effects of mutations. Our hope is that the computational results can be used to provide guidance to lab experiments when designing new proteins. Here, we report a novel, structure-based computational protocol for fast virtual mutagenesis of protein complexes. Rather than treating the protein at a fixed protonation state and calculating the mutation energy as a single value across different solution pH, our method takes into account the protonation state of titratible residues and reports the mutation energy as a function of pH. The electrostatic contribution of the mutation energy is derived from the fractional protonation of titratible residues by integrating over the proton binding isotherms.^11^ The same approach was applied by others in the previous studies to model pH-dependent protein stability,^12^ protein-DNA,^13^ protein–protein binding affinity,^14^ and cooperativity of ion binding.^15^ To our knowledge, this is the first study in which a pH-dependent model has been applied to calculate the effects of mutation on binding affinity. The method is implemented in the recent version of Discovery Studio.^16^

## MATERIALS AND METHODS

### General theory

The changes in binding affinity as a result of a mutation, 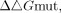 are calculated as the difference between the binding free energies, 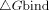, of the mutant and wild type.


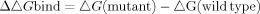
(1)

where 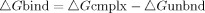. Note that negative values of 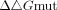 correspond to a stabilizing effect of the mutation and *vice versa*. Following a number of recently published structure-based models,^3^,^4^,^13^ the energies of the complex and unbound states, 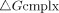 and 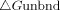, are approximated by a sum of a few interaction energy terms.


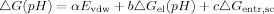
(2)

where *a, b,* and *c* are empirical weighting parameters, *E*_vdw_ is the van der Waals energy, 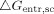 is the entropy term for the cost of reduced side-chain flexibility, and 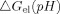 is the pH-dependent electrostatic interactions where the protein ionization characteristics are calculated using the same method as in our previous work.^17^

The free energy of binding between two molecular partners *A* and *B* is related to the equilibrium association constant *K*_a_ as 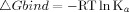 for the reaction *AB* = *A* + *B*.

In most of existing physics-based approaches (e.g., MM/FDPB^1^ or LIE^2^ methods) the binding free energy terms necessary to calculate 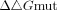 are evaluated using three separate calculations:


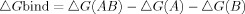
(3)

where the energy terms for the unbound partners *A* and *B* are calculated separately because of technical constraints from the linear dimension of the FDPB grid or from the water box (LIE).

Taking advantage of the pair-wise Generalized Born approximation^18^ in CHARMM GBIM method,^19^,^20^ the calculations are reduced to two sets:


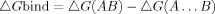
(4)

where the unbound state 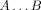 is modeled simply by separating the binding partners by a large distance (e.g., 500 Å).

To illustrate the general difficulties in calculating the binding free energy of a protein complex, let us assume, as an approximation, that the possible structures of binding partners in bound and unbound states are represented by sets of discrete conformations *R*_*j*_. Since the changes in electrostatic interactions could be a key contributor to binding free energy, and in turn, they depend on the ionization of acidic and basic groups, the titratable residues are represented by the possible states of protonation, *X*_*i*_.^21^ The free energy terms of bound and unbound states could be derived from the corresponding partition sums of all microstates:


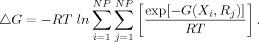
(5)

where NP = 2^Ns^ is the number of possible protonation states of *N*s titratable groups, and NC is the number of all possible conformations. Because the total number of all possible states arising from the multiple conformations and protonation states is huge, various approximations are made in the computational approaches that completely or partially omit the treatment of the multiple titration states or the protein conformational flexibility. Based on the level of approximation in the treatment of the combinatorial problem, we classify all methods, which are used or could be used in modeling the effects of the mutations, into four general classes:

1. NP = 1, NC = 1: This approximation uses a single protonation state and a single conformation as input for the energy calculations. Some well known programs such as Robbeta^3^ and FoldEF^22^ belong to this class, which calculate the binding free energy as a combination of forcefield energies and additional empirical energy terms. An earlier Discovery Studio^16^ protocol also belongs to this class and it will be described below.
2. NP = 1, NC > 1: The second approximation also neglects the treatment of multiple states of protonation, but model proteins as flexible structures. It is used in the most rigorous and computationally expensive approaches such as free energy perturbations or methods that use ensemble averages over MD trajectories such as MM/PBSA,^1^ LIE,^2^ or conformational sampling algorithms (CC/PBSA^4^).
3. NP » 1, NC = 1: As with the first class, this level of approximation neglects the conformational flexibility, but takes into account the equilibria of proton binding. Our method presented here belongs to this class.
4. NP » 1, NC ≫ 1: This approach considers both the multiple protonation states as well as the conformational flexibility of the protein. It should be the ultimate goal of future development.

Regarding the third and the fourth approaches, we were unable to find any computational methods in the literature that rigorously reports the pH dependent mutation energy terms. Instead, to improve the predictions when the standard ionization model fails, some authors^2^,^4^ model the titratable residues in their neutral charge state and add a simplified correction term of 1.36|pKa-pH| kcal/mol to 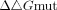. However, this approach completely ignores the cooperativity of the proton binding that in many cases is critical for the titration properties of complex systems with multiple ionization sites, as proteins are. It is not applicable for all pH values and needs other calculations or assumptions to estimate the ionization properties in the bound and unbound states. In the attempt to fill this gap, we developed a new computational protocol that automatically calculates 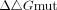 as a function of pH, described below as model MPH. For this purpose, we combined two existing computational components in Discovery Studio. The first component is for fast calculation of 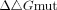 that has been developed in the “traditional” approximation using a single protonation state (model M0). The second component was developed earlier to predict the proton binding equilibria at given pH and used by another Discovery Studio protocol “Calculate Protein Ionization and Residue pK.”^17^

### Energy calculations

#### Model M0

The free energy terms are approximated with the sum of a van der Waals term, 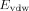 and an electrostatic term, 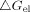 that represents the polar contribution of both the intramolecular and protein solvent interactions as described below. Two additional energy terms are added, a solvent dependent, 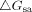 term for the non-polar contribution of solvation energy, and an empirical entropy term to account for the changes in the side-chain flexibility:


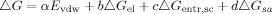
(6)

Similar to several existing methods,^2^–^4^ the empirical weighting coefficients *a, b, c,* and *d* are introduced to improve the fit with experimental data. All energy terms are calculated using CHARMm^23^ and the method is developed as program modules written in CHARMm scripting language. *E*_vdw_, 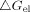, and 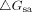 are standard CHARMm energy terms calculated using the Momany and Rone forcefield.^24^ The GBIM CHARMm module^20^ is used to calculate the electrostatic term, which extends the functionality of the method to membrane proteins. The total electrostatic contribution, 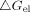, is calculated as:


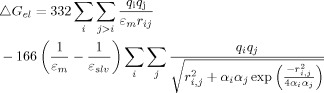
(7)

where *q*_i_ are the atomic charges, *α*_i_ are the effective Born radii, and *ε*_m_ and *ε*_slv_ are the dielectric constants of the molecule and the solvent, respectively. The contribution of side-chain entropy *S*_SC_ is approximated as:


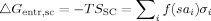
(8)

where *sa*_*i*_ is the percentage side-chain solvent accessibility of residue *i* in the folded state, σ_i_ is the average entropic cost to bury a residue in a folded structure, and the summation is taken over all amino-acid residues. The σ_i_ values can be taken from one of the available empirical entropy scales^25^,^26^ and the results in this study are obtained using the data from Ref. 26. Instead of a linear dependence^25^ on *sa*_*i*_ we suggest a sigmoid function *f(sa)*_*i*_ that ranges between 0 for entirely exposed to 1 for entirely buried residues:


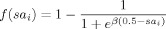
(9)

#### Model MPH

The main difference between the M0 model and the new, pH-dependent model is that the electrostatic term 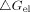 and 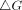 are calculated as a function of pH respectively, while the other terms are calculated in exactly the same way, as in M0:


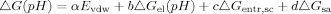
(10)

To evaluate the pH dependent 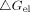 (pH) term we implemented a method, based on the integration over the binding isotherms.^11^ Similar approaches have been used to model pH-dependence of protein stability,^12^,^27^,^28^ the protein-DNA interactions,^29^ protein-ligand interactions,^17^ and cooperativity of ion binding.^15^ However, no computational tool has been reported to calculate the full scale of pH-dependent energy differences resulted from the mutations of amino-acid residues.

In the MPH model, the electrostatic contribution is calculated as:


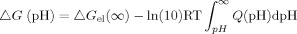
(11)

where Q(pH) can be either the average number of bound protons or the total charge. The electrostatic free energy is conveniently referenced to the energy of completely deprotonated state 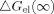.^12^ The model of deprotonated state used to calculate 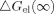 is constructed by assigning the corresponding partial charges to the atoms of all titratable groups. Q(pH) is derived from the fractional protonation of titratable residues:


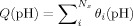
(12)

The calculations of θ_i_(pH) as well as all other pH related properties of wild type and mutant structures are carried out as described in a previous Discovery Studio method to calculate protein ionization.^17^ It is based on GBIM CHARMm calculations combined with the IMC iterative mobile clustering approach^30^ to treat the combinatorial problem of multiple protonation states. Another difference from M0 method is that the CHARMm GBIM module is used only to calculate the effective Born radii, and the electrostatic energy terms used in the calculations of 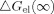 and θ_i_(pH) are carried out by a separate C++ program that extends the method by including the effect of ionic strength *I.*^31^

In addition to mutation energy terms, the MPH-based protocol also reports the predicted p*K*_a_ values, the fractional protonation of titratable residues for the wild type and mutants in both the bound and unbound states, the mutation energy at the specified pH, and the corresponding titration curves and the pH-dependent electrostatic contribution to the binding free energy.

### Modeling of the mutant structures

Both M0 and MPH methods use a module written as a CHARMm script to generate and optimize the structures of the mutants. The construction of the mutant structure includes a sampling algorithm that is similar to ChiRotor^32^ that searches for optimal conformation of the side-chain of the mutated residue at the fixed backbone.

### Implementation details

The method is implemented for both CHARMm and CHARMm Polar H (hydrogens) forcefields.^24^ The results shown in this study were obtained using CHARMm Polar H.

The methods use a number of CHARMm scripts, C++, and Perl program modules wrapped in a single Accelrys Pipeline Pilot protocol “Calculate Mutation Energy (Binding).” The input list of the mutations is generated automatically from the list of selected residues and amino acid types of the substitutions. The relationship between CPU time for a single mutation and the size of the proteins is almost linear. For a medium size protein of about 200 residues, it takes about 30 sec per mutation using M0 approximation and 1.5 min using MPH. In addition, a coarse grain parallelization implemented in the protocol allows automatically and easy distribution of the individual mutations to a large number of available processors and servers.

The use of the Generalized Born solvation model in combination with IMC approach makes the calculations fast and applicable to very large systems (e.g., more than 1000 sites of titration), and unlike many grid-based methods, is independent on the size of interacting molecules. Also, the computational protocol is applicable to membrane environments, and besides protein–protein complexes, it can be used to study the effect of mutation on the binding of ions and other compounds such as organic ligands or DNA/RNA molecules.

### Homology model of human Fc and FcRn

Given that the sequences of the Fc domain of human IgG are very similar, we choose the subtype 1 as input to the homology model. For FcRn, human sequence from pdb structure 3m17 is used which includes the beta-2-microglobulin domain. The sequence alignment of Fc-FcRn complex between murine (pdb code 1i1a) and human was generated using multiple sequence alignment method in Discovery Studio 3.5. The sequence identities between the murine and the human for Fc and FcRn domains are 64 and 70% respectively. Twenty homology models are created using MODELER^33^ implemented in Discovery Studio and the model with the smallest violation to the homology restraints are selected. Further analysis of the models reveals that some of the interface residues have highly conserved side-chain conformation and others highly variable. The residues with conserved side-chains mostly have identical residue type between murine and human and those with the highly variable side-chain have different residues between the two organisms. For those that are highly variable, additional refinements are carried out using the CHARMm based side-chain optimization method, ChiRotor.^32^ The resulting model is then used for all the mutation energy calculations.

## RESULTS

### Parameterization of the method and alanine scanning tests

The weighting coefficients *a, b,* and *c* were determined based on matching the mutation data from one protein (1a22 in Supporting Information Table S1) and verified on a set of experimental data for 380 mutations to alanine. Briefly, the alanine scanning results (Supporting Information Table SII) showed a state of the art accuracy with 0.77 kcal/mol unsigned error and a correlation coefficient *R* = 0.72. For a previous, pH-independent variant of the model, the best correlation with experimental 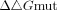 were found at α = b = 0.5 and c = 0.8. For pH-dependent model represented by Eq. (2), the best parameter values were found at a = 0.5, b = 1 and c = 0.8. It is notable that after adopting a physically more consistent electrostatic model, there was no need to scale the electrostatic energy and *b* is set to 1.

In our previous work on protein ionization,^17^ the most accurate p*K* predictions were achieved at values of intramolecular dielectric ε_m_ constant equal to 10÷11. In this study, we are using the same electrostatic model. To keep the calculations of all electrostatic energy terms consistent with the calculations of the ionization properties, the dielectric constants in Eq.(7) were set to ε_m_ = 10 and ε_slv_ = 80, except for the mutations of the P1 residue of serine proteinase inhibitor. This residue is buried deep inside the protein complex, therefore a slightly decreased value of ε_m_.= 8 was used to account for the less polarazible surrounding.

A more detailed analysis of alanine scanning experiments and the general accuracy of the method will be presented elsewhere and this study will focus on cases where the effect of pH on the binding can be important.

### Serine protease

**Figure 1:**
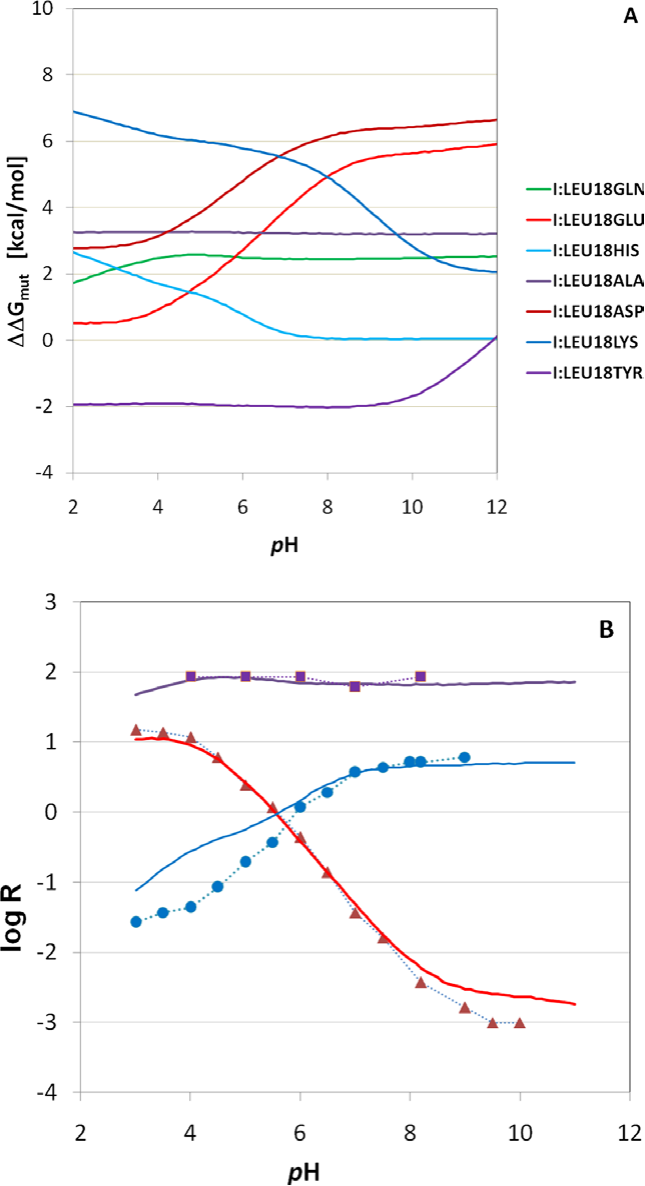
Binding of OMTKY3 inhibitor to proteinase B. **A**: 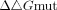 (pH) calculated for the substitutions of P1 residue Leu18 by several amino-acid types. The input data was the atomic coordinates of the OMTKY3 and protease B complex (pdb code: 3sgb). **B**: pH-dependence of logR, derived from the calculated mutation energies shown in Fig. 1A. *R* is the ratio of the binding constants *K*_a_(Glu18)/*K*_a_(Gln18) (red line), *K*_a_(His18)/*K*_a_(Gln18) (blue line), and *K*_a_(Leu18)/*K*_a_(Gln18) (green line). The triangles, circles, and squares represent the experimental log*R* values, obtained in the study of pH dependency of OMTKY3 binding to proteinase B.^6^ Note that the log*R* values for Glu18/Gln18 and Leu18/Gln18 are derived directly from 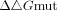
(pH), while the *K*_a_(His18)/*K*_a_(Gln18) curve is shifted down to compensate for a ∼1.5 kcal/mol nonelectrostatic over-stabilization of His18 mutant.

One of the most extensively studied systems on the effect of mutation on binding affinity is the canonical inhibitors of serine proteases where the primary specificity determining residue (P1) has been mutated to almost all standard amino acid types. In addition, a few mutations of the P1 residue of turkey ovomucoid third domain (OMTKY3) that binds to proteinase B have been measured experimentally in a wide pH interval.^6^ This served as a test case for us to assess how good our method can predict the pH-dependent mutation energy. As seen in Figure1, the calculated binding constant ratios Leu/Gln, His/Gln, and Glu/Gln agree very well with the corresponding experimental results with regard to the pH-dependent behaviors, which leads us to believe that the computational protocol for pH-dependent mutagenesis is relevant and motivated us to investigate further. The results from the experimental and *in silico* data shown in Figure 1 demonstrate that the effect of certain mutations can depend on pH in a nontrivial way and even a single amino-acid substitution could result in significant changes of up to 3–4 orders of magnitude in binding affinity when changing the solution pH.

### pH-selective binding of IgG to neonatal receptor

**Figure 2:**
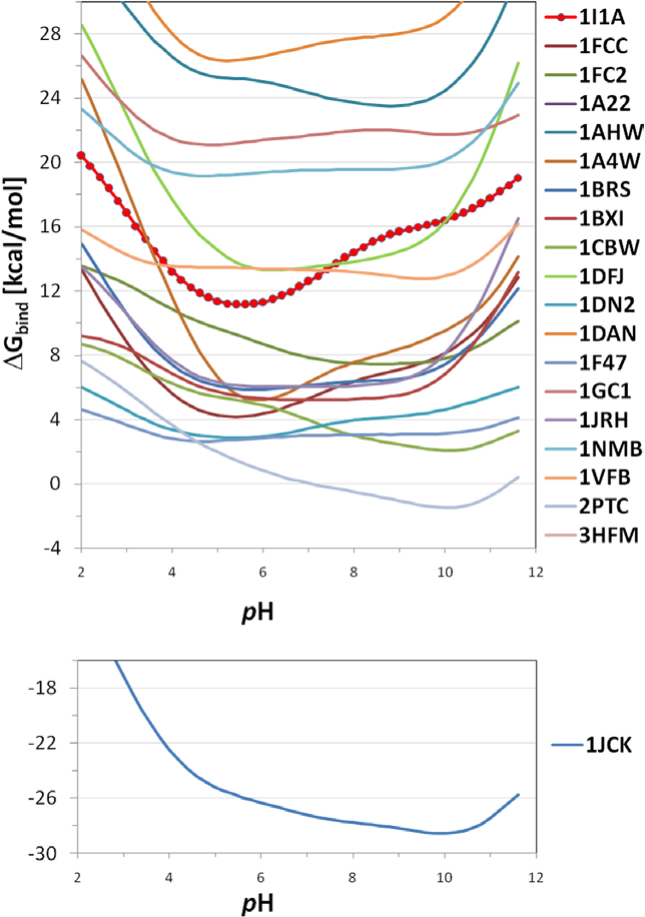
The binding energy as a function of pH calculated for Fc-FcRn complex (red circles), compared to the calculated binding curves for 19 protein complexes from Supporting Information Table S1.

The interaction of immunoglobulin G (IgG) with neonatal receptor (FcRn) is a striking example of a natural design of proteins with pH-selective binding, which is critical for the biological function^7^ of IgG. The ability of IgG (via Fc domain) to strongly bind to FcRn at low pH (<6.5) and to be effectively released at physiological pH (7.4) is the key to its long half-life in serum and offers the opportunity to engineer antibodies with longer or shorter half-lives depending on the goal of the application. Figure2 shows the pH-dependent electrostatic contribution to the binding energy of the Fc-FcRn complex calculated using Eq. (3) compared to pH binding profiles of 19 protein complexes from Supporting Information Table SI.

While binding energy curves for most of the proteins are on plateau or show a slight increase from pH 6 to pH 8, the Fc-FcRn complex demonstrates a steep increase in full agreement with its physiological behavior.^7^ The calculated binding free energy increases 2.2 kcal/mol from pH 6 to pH 7.5, which is in line with the experimental^34^ *K*_*d*_ ratios, corresponding to 2–3 kcal/mol binding free energy difference, or 50–100 times stronger binding at pH 6. Only two other complexes out of the 19 show a similar, but less steep slope. One is also an IgG (1fcc), in complex with protein G and the result is consistent with known experimental^35^ data that shows a pH dependent binding of protein G with monoclonal antibodies.

### Hotspots for IgG and FcRn binding and pH-selectivity

**Figure 3:**
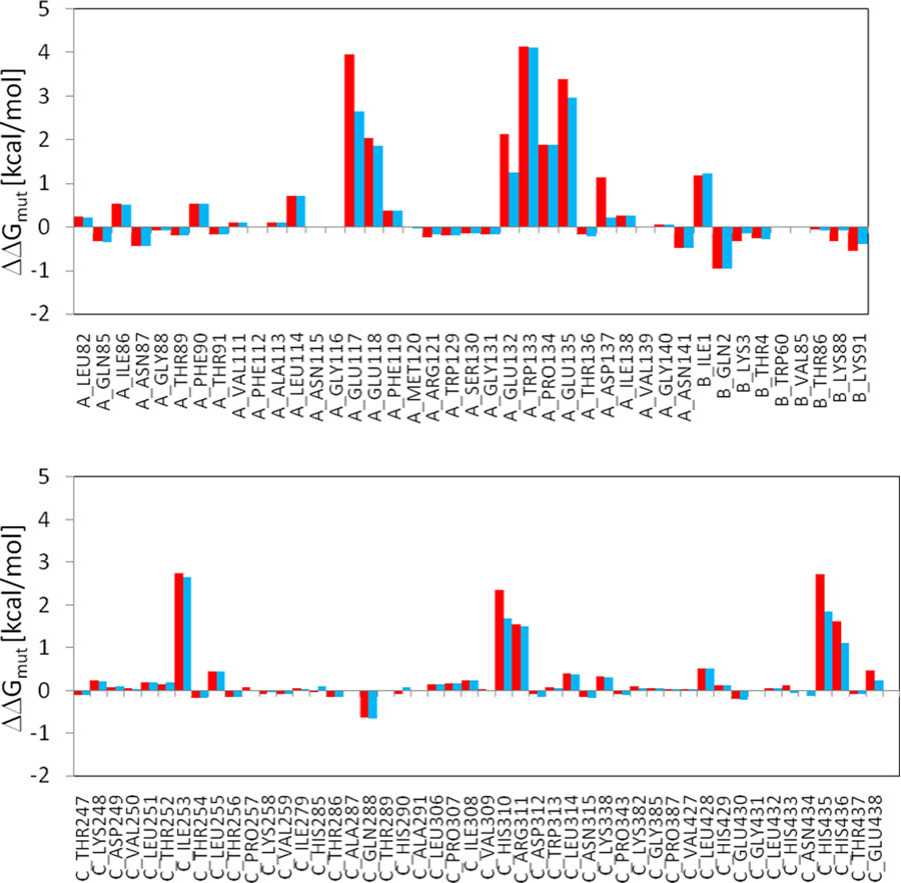
The free energy of mutation, 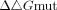, at pH 6.0 (red bars) and pH 7.5 (blue bars) extracted from the results of alanine scanning of murine Fc-FcRn interface residues. The IDs A and B correspond to FcRn heavy chain and FcRn microglobulin parts. C corresponds to IgG Fc fragment that is in contact with FcRn.

After we established that the method can generate pH-dependent binding profiles reliably, we took further analysis to identify the residues on the binding interface which are responsible for binding affinity as well as for pH-selectivity. The input for our calculation was the X-ray structure (pdb code: 1i1a)^36^ of the Fc-FcRn complex from murine. First, an alanine scanning of all residues from Fc-FcRn binding interface was carried out and 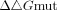 were calculated from pH 2 to pH 12. The interface residues are defined as the residues with interatomic distances within 8.0 Å between Fc and FcRn. The full pH profiles of binding energy differences are shown in Supporting Information Figure S2. Figure3 shows the 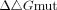 values at pH 6 and 7.5 corresponding to the two physiological environments of interest.

With a relatively short calculation (about 3 h using a single CPU medium power desktop PC), all major players responsible for the binding, as well as for pH selectivity, are automatically revealed and the results are in full agreement with the findings from multiple experimental studies reviewed in Ref. 7. The acidic residues Glu117, Glu132, and Asp137 from FcRn heavy chain and their IgG interacting partners His310, His435, and His436 are among the residues mostly responsible for the stabilization of the complex, and for the reduced binding affinity at pH 7.5. The pH-dependent binding behavior can be seen from the reduced destabilizing effect of alanine substitution at pH 7.5. The alanine scanning also shows a strong stabilizing and expectedly pH-independent contribution to binding from the hydrophobic residues of FcRn Trp133 and Fc Ile253. The magnitude of the calculated destabilizing effect of 2.3 kcal/mol for His310Ala and 2.7 kcal/mol for Ile253Ala at pH 6 are in good agreement with the experimental data^34^ of roughly 3 kcal/mol when mutating those residues to alanine. The results shown in Figure3 demonstrate that a simple *in silico* alanine scanning can reveal the hotspots for pH-selectivity and for general binding affinity, which would have otherwise required more costly and time consuming experimental work. A few more mutations of the FcRn interface residues are in good agreement with the experimental data^37^ and the results can be found in Table I.

**I:** The Effect of Mutations of Murine FcRn, Compared to Experimental ΔΔ*G*_mut_ Values

	ΔΔ*G*_mut_ [kcal/mol]	

Mutation	Calculated	Experimental^37^
β Ile1Ala	1.2	>2
β Gln2Ala	−0.9	−0.5
Trp133Ala	4.1	>4.0
Asp137Asn	2.2	>4.0
Asp117Ser	4.6	>4.0
Glu132Gln	3.6	
Glu135Gln	3.6	
Glu132Gln and Glu135Gln		>4.0

The structure of the human Fc-FcRn complex is not available experimentally; however, the sequence identity of human Fc to murine, and the corresponding human FcRn to murine FcRn are both over 60%. We used the murine structure as template to generate homology model of human Fc-FcRn complex. The alanine scanning results of the homology model (Supporting Information Fig. S8) reveals the hotspots of binding as well as pH-selectivity in full agreement with experimental observations. Similar to the results from murine complex, the hotspots for binding affinity on human Fc interface are Ile253, His310, and His435 with the latter two exhibiting pH-selectivity. His436 in murine IgG is mutated to Tyr436 in human which remains as a hotspot for binding due to the similar aromatic side-chain, however, the pH-selectivity is lost.

### *In silico* design of pH-selective binding of IgG to FcRn

**Figure 4:**
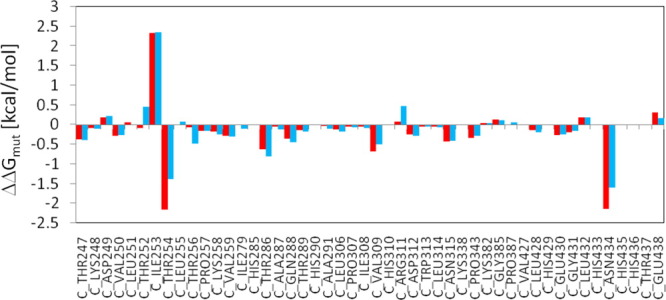
The free energy of mutation, 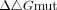, at pH 6.0 (red bars) and pH 7.5 (blue bars) extracted from the results of histidine scanning of murine Fc residues from FcRn interface (Supporting Information Fig. S3).

While the results of alanine scanning identify the residues with major contribution to the binding energy and pH-selectivity, it is insufficient for the prediction of mutations that can improve the pH-dependent binding behavior. Therefore, in an attempt to find prospective IgG mutations that could prolong the antibody half-life, we used a more comprehensive search strategy by mutating the murine IgG interface residues to several amino-acid types with different ionization properties. The Supporting Information Figures S3–S7 show the results of mutating IgG interface residues to the titratible residues His, Lys and Glu and hydrophobic Leu and Phe. As expected, mutations with the strongest pH-dependency between pH 6 and 7.4 came from the results of histidine scanning (Fig.4). Two separate single mutations, Thr254His and Asn434His, show considerably stronger binding at pH 6, but moderately improved binding at higher pH. Three other mutations, Thr252His and Arg311His, Thr254Glu (Supporting Information Fig. S4) also show pH-selective profile and has reduced binding affinity at pH 7.4 with no significant effect at pH 6. The latter three mutations may not improve the half-life of IgG in serum directly; however, combining them with stabilizing mutations which are pH-insensitive can potentially lead to improvement of the serum half-life.

There have been very few successful attempts to increase the half-life of murine antibody in serum and one of them is a triple mutation on murine Fc domain, Thr252Leu, Thr254Ser, Thr256Phe.^38^ The authors find that this mutation stabilizes the complex by ∼ 0.8 kcal/mol at pH 6 relative to the wild type. Interestingly, according to our calculations, the net cumulative effect of the three single mutations Thr252Leu, Thr254Ser, and Thr256Phe (LSF) at pH 6 is also stabilizing by about 1.2 kcal/mol 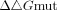 are −0.5 and −1.3 kcal/mol for Thr252Leu and Thr256Phe as seen in Supporting Information Figures S6-S7 and 0.6 kcal/mol for Thr254Ser. Moreover, the calculations provide a reasonable explanation for the neutral effect observed experimentally of another triple mutation Thr252Val, Thr254Ser, and Thr256His (VSH). Considering the Thr252Val has similar effect as Thr252Leu, less stable effect of the VSH mutation vs. LSF can be explained by characteristics of histidine. Here, the electrostatic repulsion and desolvation penalty of charged His256 at low pH offset the van der Waals stabilization (Fig.4) effect which leads to a neutral effect of Thr256His mutation at pH 6.

On the other hand, given the importance of half-life of therapeutic antibody in human system, several experimental attempts have been made to identify mutations that will improve the binding of Fc to FcRn and prolong the serum half-life of human IgG. Some level of success has been achieved and so far the best result is observed in the triple mutation M252Y/S254T/T256E (YTE) of an engineered monoclonal antibody MEDI524^8^ which improved the half-life of the human IgG in serum by 4 folds. The net cumulative effect of the three single mutations is −0.7 kcal/mol at pH 6 by our calculation which is stabilizing, but weaker, compared to experimentally observed 10-fold increasing in binding affinity, corresponding roughly to −1.4 kcal/mol in mutation energy. Both our calculation and experiment agree that the YTE triple mutation is not pH sensitive and the mutant keeps the same pH-dependent binding profile as the wild type.

Almost all of the mutations experimentally studied so far are not pH-sensitive. In another words, the mutations increase/decrease binding of Fc to FcRn to the same extent under pH 6 and 7.4. As a result, this imposes a top limit of how much we can improve the half-life of antibody in serum since strong binding at pH 7.4 will lead to fast clearance of antibody. Here based on our analysis, we propose a slightly different general strategy for the design of IgG or other proteins with improved affinity at a specific pH and retained or reduced affinity at another pH.

**Figure 5:**
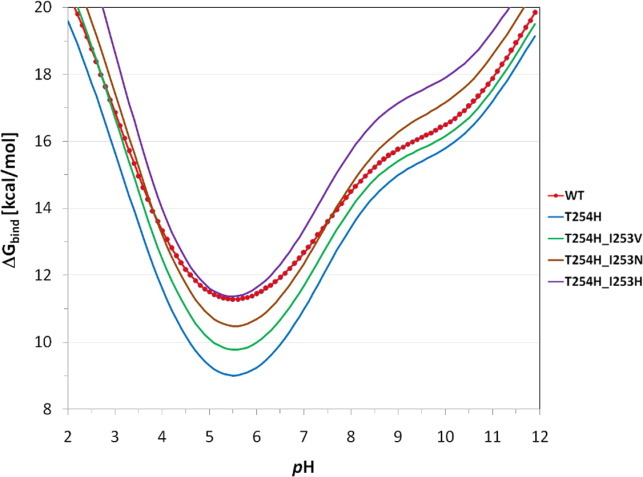
Free energy of binding of murine Fc to FcRn calculated as a function of pH for variants of mutant structures of the Fc fragment. Note that the wild type curve represents only the electrostatic contribution, while the binding energies for the mutants include the van der Waals and entropy energy differences between the mutant and the wild type.

- Perform a set of amino-acid scanning experiments to find one or more mutations with the desired pH shape of 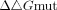 (pH) within the pH interval of interest (here pH 6–7.5). In our study, the histidine scanning identified two pH-selective mutations Thr254His and Asn434His from murine Fc that show a significant improvement of the binding at pH 6 and moderate increase in binding at pH 7.4. Because Thr254His appears to be more selective, its structure has been retained for a further search.
- Find a second, pH-insensitive compensatory mutation that will shift the ΔG_bind_ curve up or down so that the binding affinity is close to desired value at pH 7.4. It can be done in many different ways. But here, using the information from the alanine scanning, we substituted Ile253 in the structure of Thr254His mutant with several types of amino-acid residues (Fig.5). The comparison of the binding energy profile of the double mutant Thr254His – Ile253Asn has a pH shape that comes very close to meeting the goals of the design. However, one of the other mutations, Thr254His – Ile253Val could also be considered because it shows ∼10 times stronger binding affinity at low pH at the price of a small increase at pH 7.5.

Similar analysis was performed for human Fc-FcRn complex based on the homology model. Interestingly, the same Thr254His mutation is also stabilizing, but less pH-sensitive (see Supporting Information Fig. S9). Another mutation, Gln311His, is stabilizing with some pH-dependency in the desired direction. However, Glu scanning results (Supporting Information Fig. S10) suggest other variants with stronger pH dependency. One of them is the His435Glu which reduces the binding affinity more at pH 7.5 than at pH 6.

**Figure 6:**
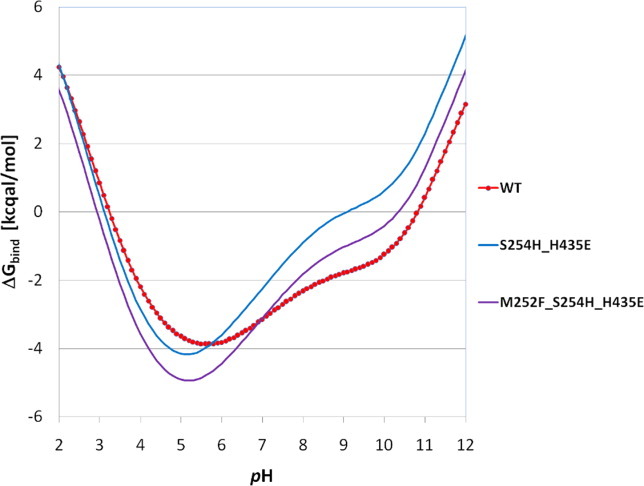
Free energy of binding of human Fc to FcRn calculated as a function of pH for variants of mutant structures of the human Fc fragment.

The double mutant, shown in Figure6, is His435Glu combined with the stabilizing mutation Thr254His. The net effect, expressed in 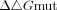 (pH) of Thr254His – His435Glu mutant is neutral at low pH with a sharp increase in binding energy between pH 6 and 7.5. Following the same approach, as for murine proteins, to shift the binding curve down, we added a third, pH-independent, but stabilizing mutation, Met252Phe, suggested from Phe scanning calculations. The triple mutation with the improved pH-selective profile (Fig.6) is a potential candidate to test experimentally for improving the human antibody half-life in serum.

## DISCUSSION

Modifying the proteins to have different binding properties with their interacting partners is the main task in protein engineering. The computational method we presented here can predict not only the mutations of a protein to improve its binding affinity with a specific partner, but also the mutations that change pH-dependent binding behavior. This method is general enough to be applied to any type of protein and in this study we are applying it to improve the half-life of monoclonal antibody IgG based on its pH-dependent binding to FcRn.

First, to validate the method, we demonstrated that we can reproduce with a state-of-art accuracy the experimental energy effects of 380 mutations from 19 proteins, including those measured at unusual pH (Supporting Information Table S2). Furthermore, the pH-dependent profile of mutating the P1 residue (Leu18) of OMTKY3 to Glu and His are well reproduced.

In addition, the study shows that with very fast and easy to set up calculations (3 h on a medium powered single CPU desktop PC), the hotspots responsible for binding, including the pH-selective binding on Fc-FcRn interface are accurately identified for both murine and human complexes. We can also match the experimentally found stabilizing mutations for the murine complex and for most of the important ones found in human complex. It is encouraging that with a high quality homology model of the human complex, the method can identify the hotspots and suggest possible mutations for improving the binding.

Recent experimental studies demonstrate that the IgG half-life in serum can indeed be prolonged by increasing its binding to FcRn^7^,^8^ at low pH in endosome. However, all the engineered antibodies produced so far also have increased binding to FcRn at normal pH in serum. This imposes an upper limit for how much we can improve the half-life of IgG in the host system.^9^,^39^ In this article, we proposed a simple strategy that combines the pH-sensitive and pH-insensitive mutations that lead to engineered proteins with desired binding affinities at different pH environments as shown in Figure5. We hope this approach can suggest mutations that can further increase the half-life of IgG in serum. With the full understanding that *in silico* predictions are not always confirmed in real experiments given that there are so many other factors influencing the binding, we hope that our results will motivate experimentalists to consider the pH-dependent computational mutagenesis as a tool in their search for new proteins. The method we presented here can perform mutagenesis scanning of the IgG residues interacting with FcRn in a few hours on a standard laptop. We believe that the method is reliable enough to be used for initial screening to find candidates for further experimental study. Besides the engineering of protein therapeutics, the method can be applied in many other areas, such as studying the effect of mutations on ligand binding, protein-DNA interactions, or for creating pH-selective protein inhibitors or enzymes for the purposes of biotechnology.
