# Supplementary material for: pH-selective mutagenesis of protein–protein interfaces: In silico design of therapeutic antibodies with prolonged half-life
Source: Proteins. 2012 Dec 12;81(4):704–14. doi: 10.1002/prot.24230 (PMC3601434; doi:10.1002/prot.24230)
Supplement: Supplementary file 1 [file prot0081-0704-SD1.pdf]

Supplementary Information for

pH-selective mutagenesis of protein-protein interfaces: *In silico* design of therapeutic antibodies with prolonged half-life

Velin Z. Spassov and Lisa Yan*

Accelrys, 10188 Telesis Court, Suite 100, San Diego, California 92121

* To whom correspondence should be addressed. E-mail: vss@accelrys.com

This PDF file includes:

SUPPLEMENTARY FIGURES (S1, S2, S3,S4, S5, S6, S7, S8, S9, S10, S11)

SUPPLEMENTARY TABLES (S1, S2)

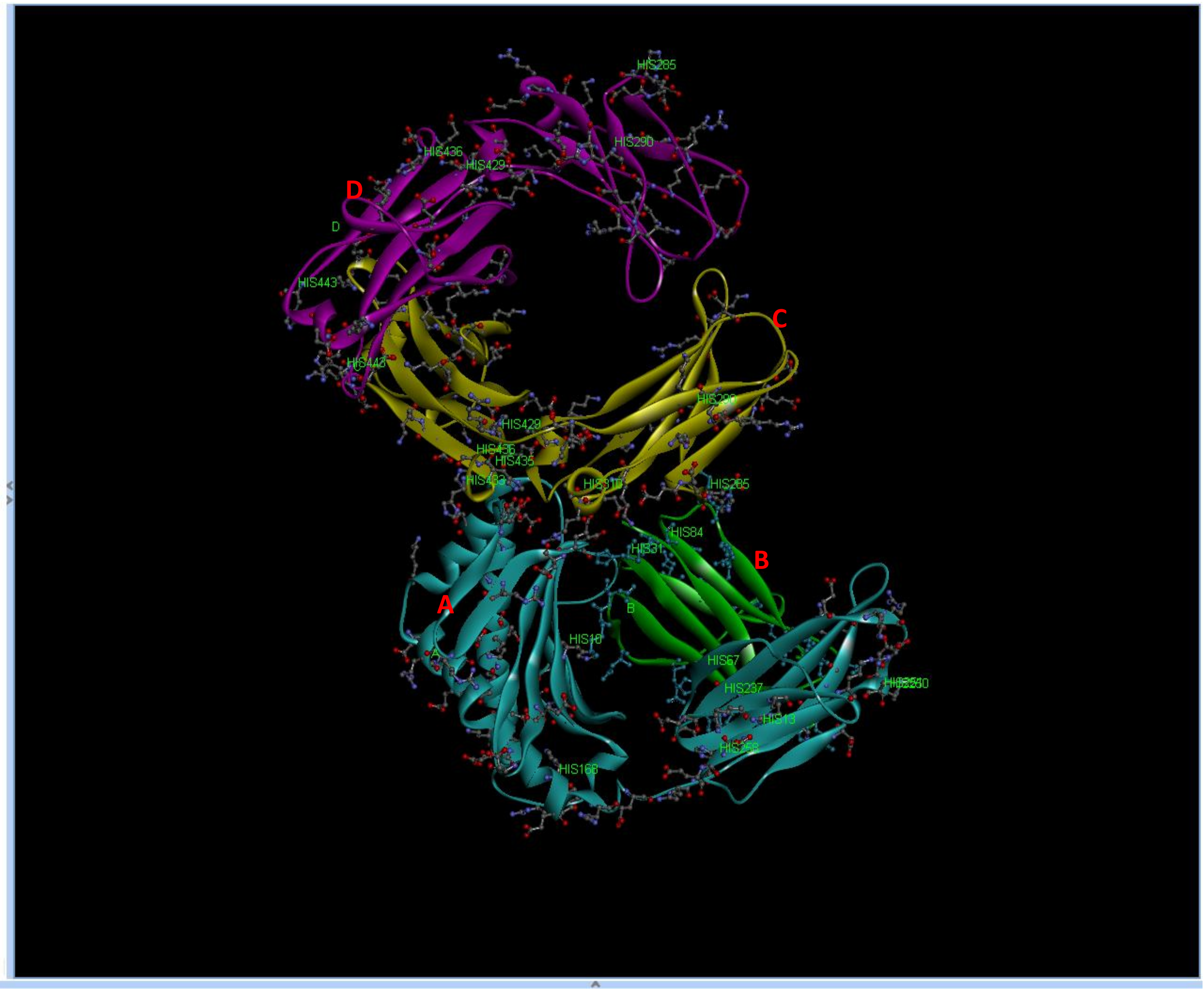

Figure S1. Structure of murine Fc-FcRn complex [20] used in calculations. FcRn receptor is present by two chains, FcRn extracellular domain (A) and β2-microglobulin (B). Immunoglobulin G Fc regions form a dimer with chains C and D.

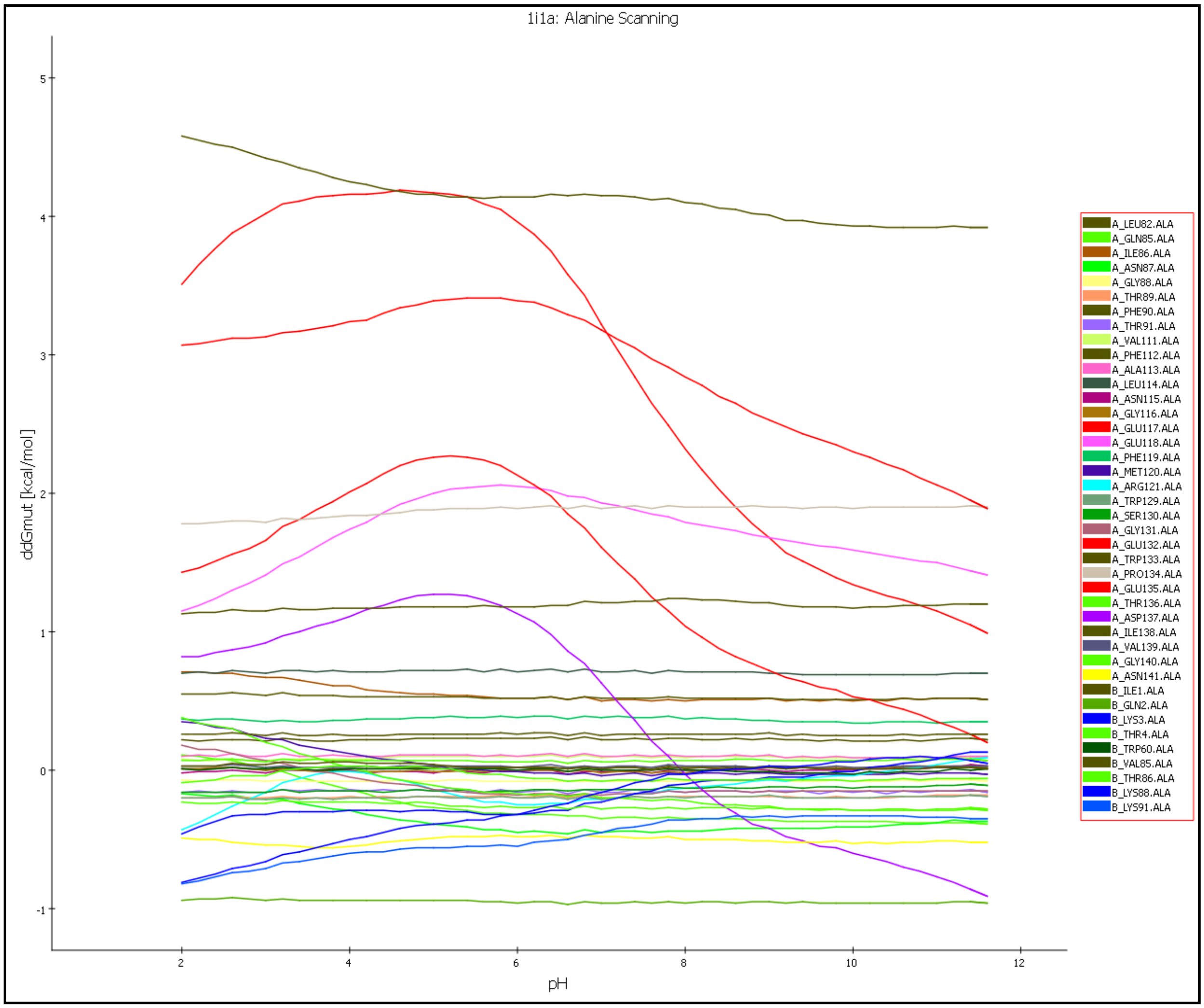

Figure S2A. pH-dependent alanine scanning of murine FcRn residues from Fc-FcRn interface. The A and B residue IDs correspond to A and B chains in 1I1A structure.

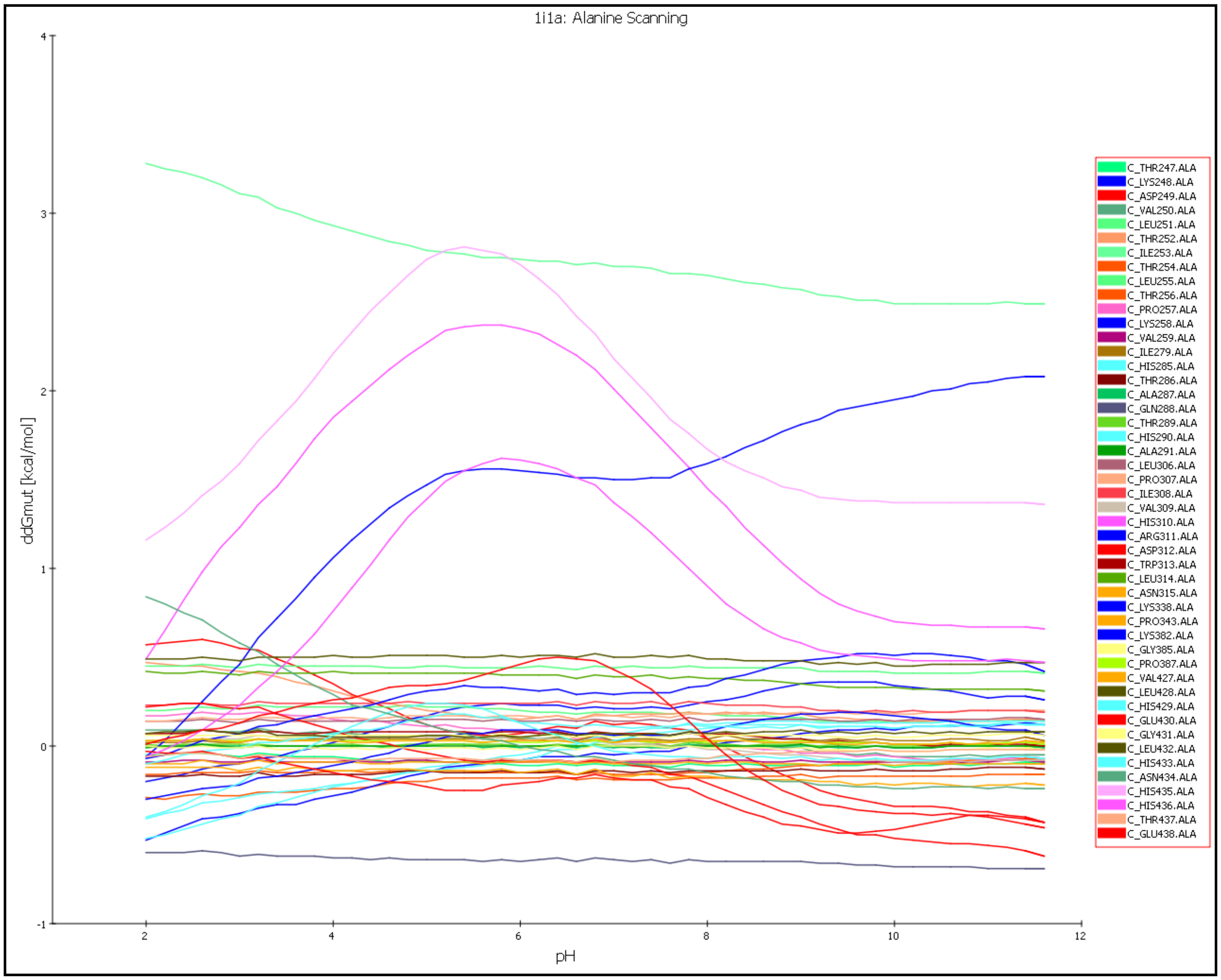

Figure S2B. pH-dependent alanine scanning of murine Fc residues from Fc-FcRn interface. C corresponds to the C chain in 1I1A structure.

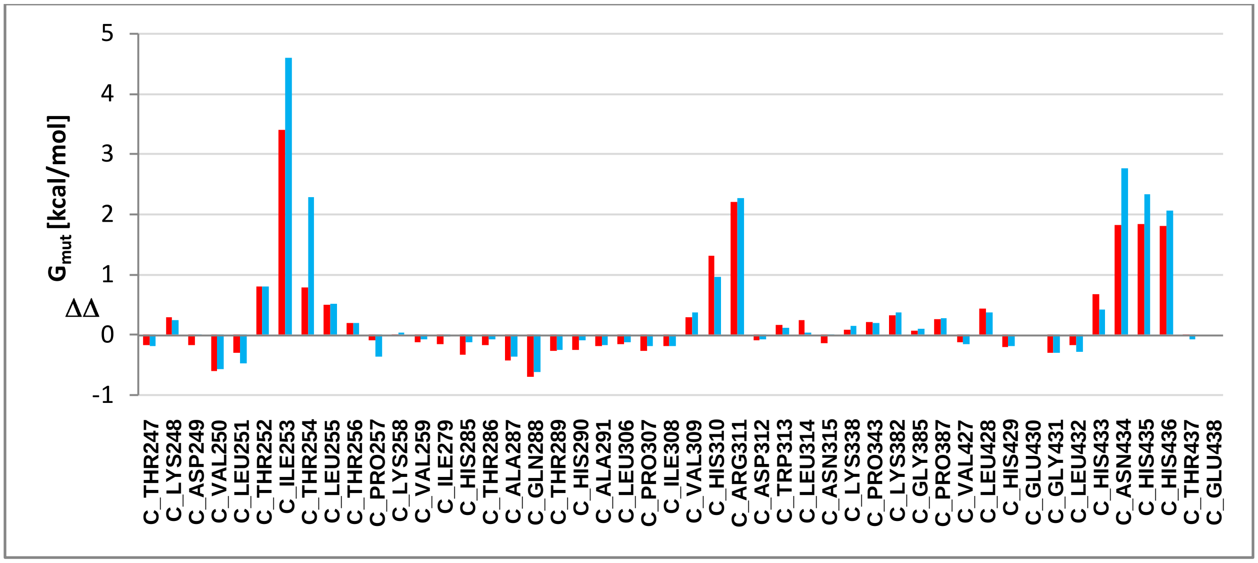

Figure S4. Selected results from the Glu scanning of murine Fc residues from the Fc-FcRn interface. The red bars represent the values of $\Delta\Delta G_{mut}$ calculated at pH 6.0 and blue bars at pH 7.5.

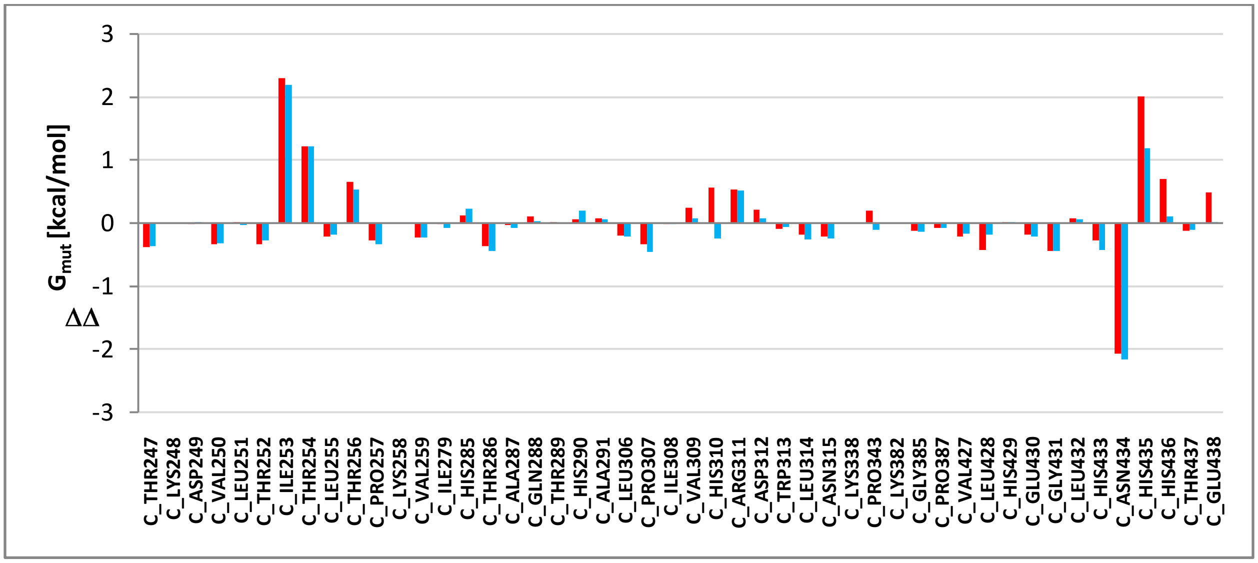

Figure S5. Results of Lys scanning of murine Fc interface residues at pH 6 (red bars) and pH 7.5 (blue bars).

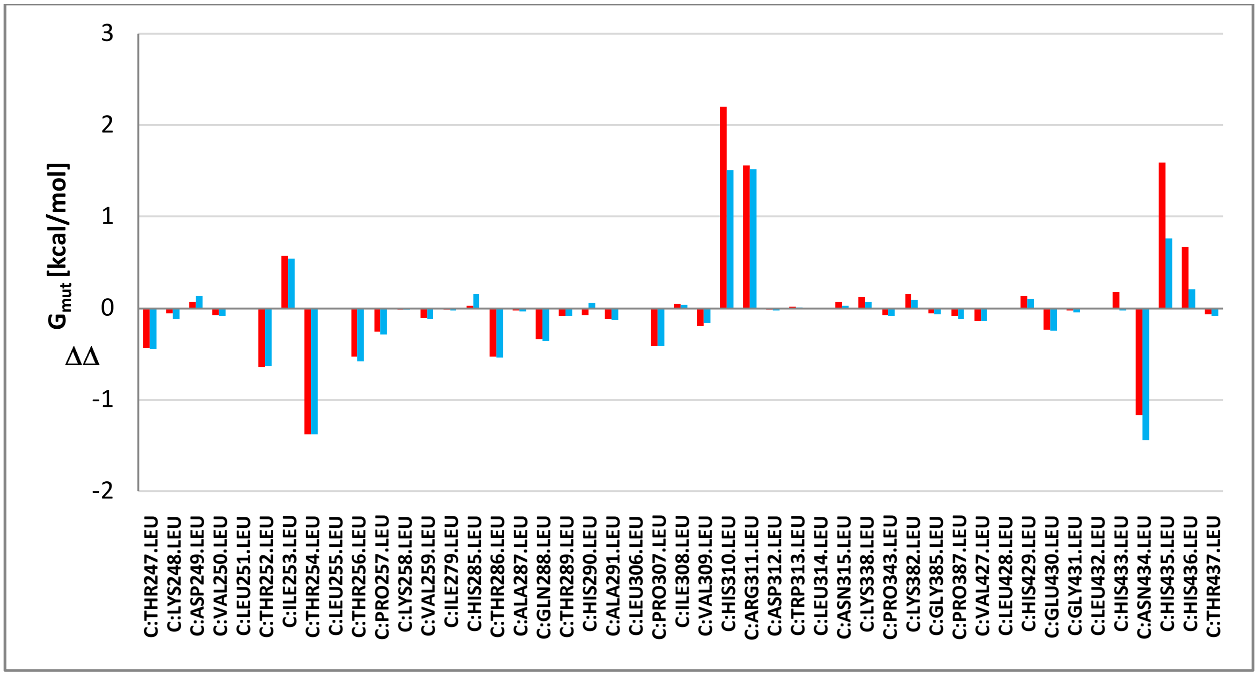

Figure S6. Results of Leu scanning of murine Fc interface residues at pH 6 (red bars) and pH 7.5 (blue bars).

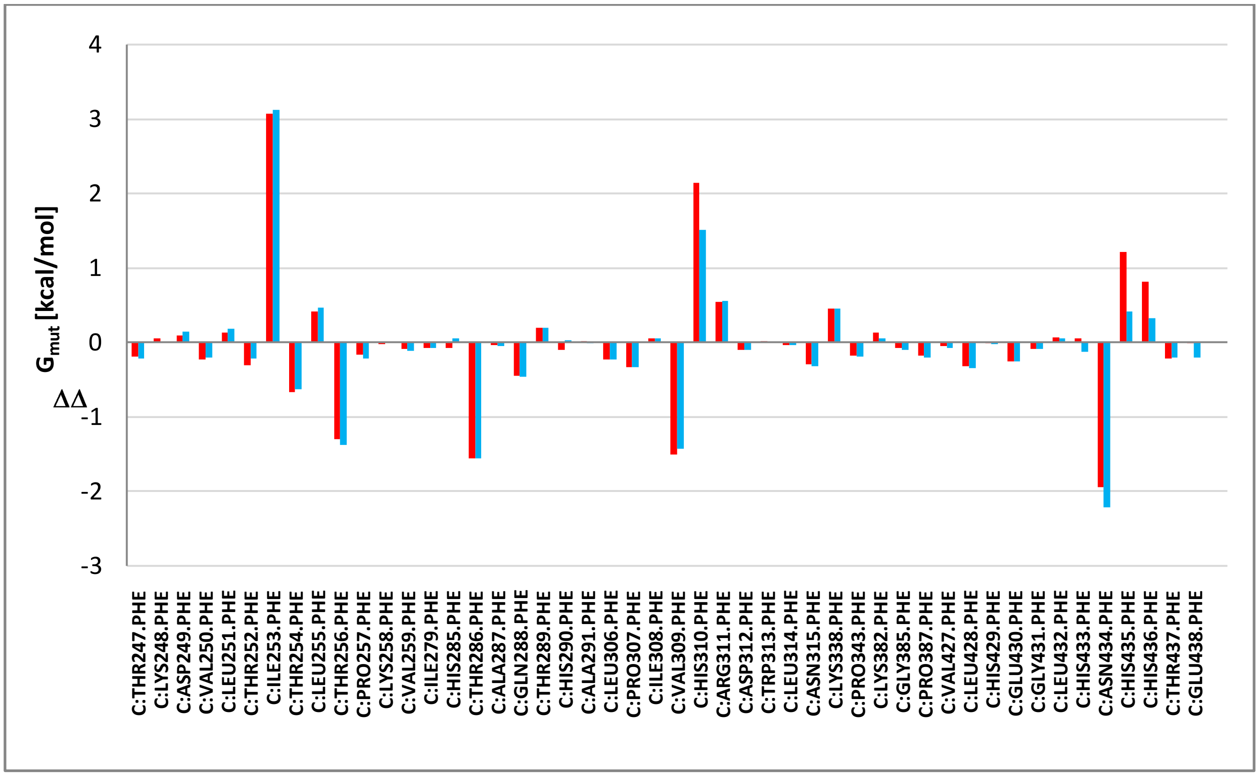

Figure S7. Results of Phe scanning of murine Fc interface residues at pH 6 (red bars) and pH 7.5 (blue bars).

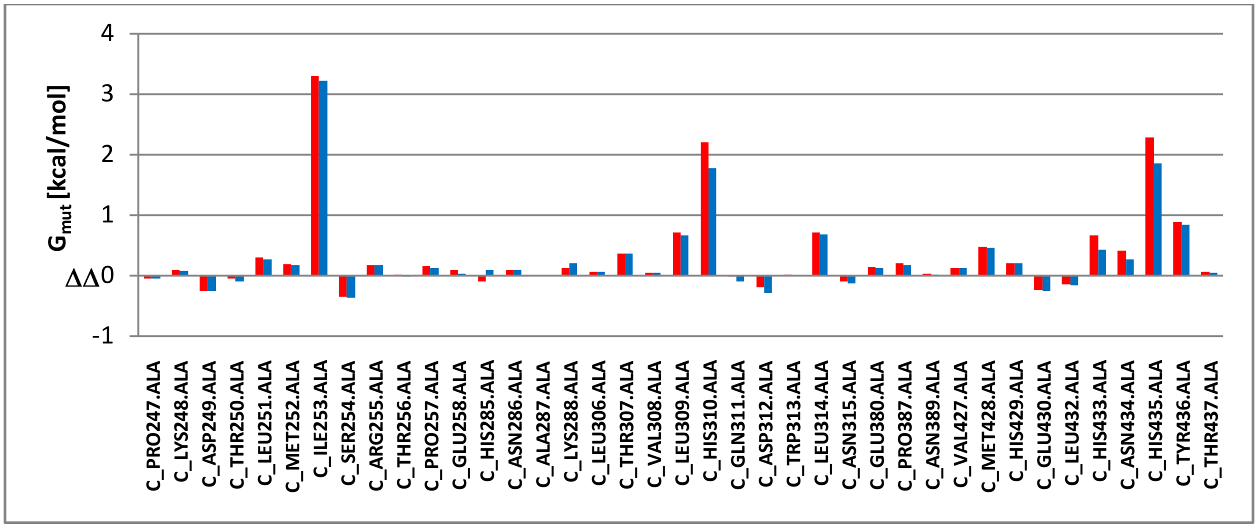

Figure S8 Results of of Ala scanning of human IgG Fc residues from Fc-FcRn interface at pH 6 (red bars) and pH 7.5 (blue bars).

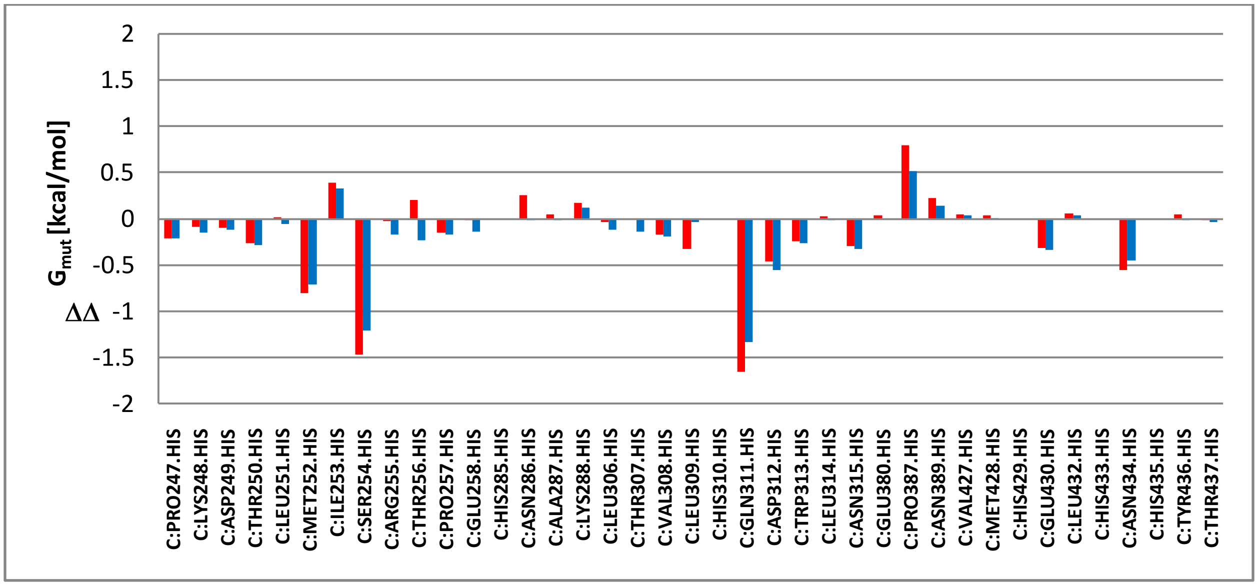

Figure S9. Results of His scanning of human IgG Fc residues from Fc-FcRn interface at pH 6 (red bars) and pH 7.5 (blue bars).

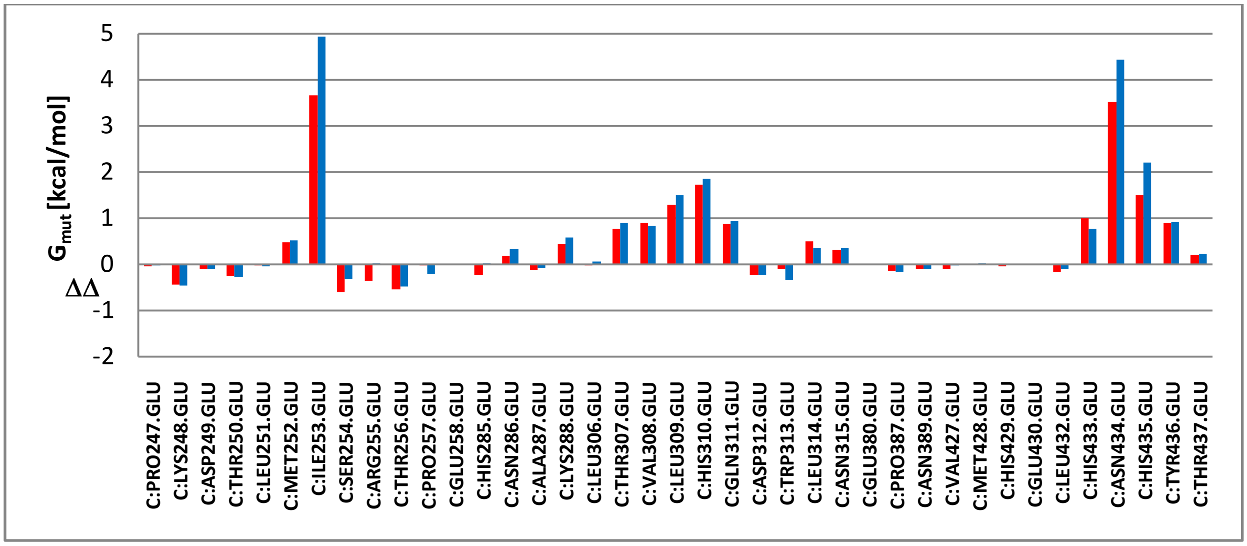

Figure S10. Results of Glu scanning of human IgG Fc residues from Fc-FcRn interface at pH 6 (red bars) and pH 7.5 (blue bars).

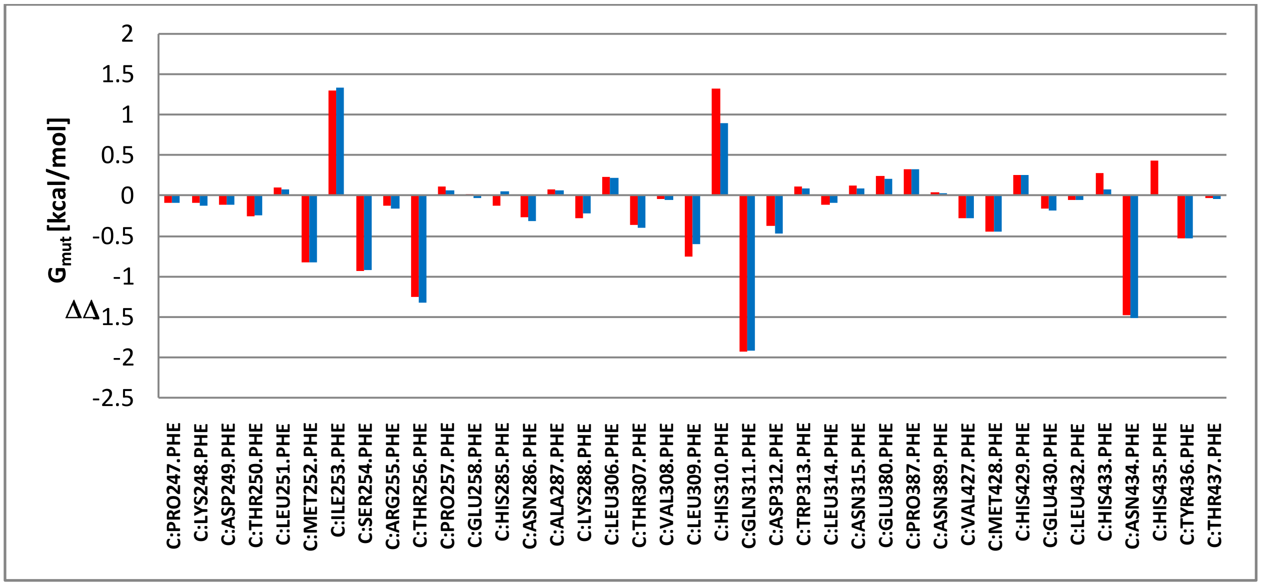

Figure S11. Results of Phe scanning of human IgG Fc residues from Fc-FcRn interface at pH 6 (red bars) and pH 7.5 (blue bars).

Table S1. Protein structures used in alanine scanning tests and in the calculations of pH-dependence of binding free energy. The selection is identical to the set used in Robbeta calculations [3].

Binding partners		PDB code	pH	Number of Mutations
hGH	hGHbp	**1a22**	7.4	65
Angiogenin	Ribonuclease inhibitor	**1a4y**	6.0	28
Barnase	Barstar	**1brs**	8.0	14
Colicin E9	DNase-Im9	**1bxi**	7.0	30
Chymotrypsin	BPTI	**1cbw**	8.4	8
Trypsin	BPTI	**2ptc**	8.0	1
Factor VIIa	Human tissue factor	**1dan**	7.4	43
Rnase A	Rnase inhibitor	**1dfj**	6.0	14
Zip A	FtsZ fragment	**1f47**	7.4	9
CD4	gp120	**1gc1**	7.4	49
S. Enterotoxin C3	T Cell receptor	**1jck**	7.4	9
Fab 5G9	Tissue factor	**1ahw**	7.0	8
HyHEL-10 antibody	Hen egg lysozyme	**1c08**	7.0	26
Immunoglobulin G	Engineered 13 residue peptide	**1dn2**	7.0	5
Immunoglobulin G	Protein A	**1fc2**	7.4	3
Immunoglobulin G	Protein G	**1fcc**	6.0	8
A6 antibody	Interferon gamma receptor	**1jrh**	8.0	31
FAB NC10	N9 Neuraminidase	**1nmb**	7.4	1
D1.3 antibody	Hen egg lysozyme	**1vfb**	7.2	28

Table S2. Results of alanine scanning of the protein structures from **Table S1.**

**PDB Code**	**pH**	**Residue**	**ΔΔG$_{mut}$ [kcal/mol]**			
			Discovery Studio **Fixed ionization**	***Discovery Studio*** **pH-Dependent**	**Robbeta [3]**	**Experiment** (data from Ref. 3)
1A22	7.4	A_ARG167	1.625	0.415	0.42	0.3
1A22	7.4	A_ARG178	1.198	0.553	0.38	2.4
1A22	7.4	A_ARG183	0.493	0.283	0.07	0.5
1A22	7.4	A_ARG64	2.862	2.452	2	1.8
1A22	7.4	A_ASN63	0.271	0.181	0.39	0.3
1A22	7.4	A_ASP171	0.663	1.048	8.73	0.8
1A22	7.4	A_ASP26	-0.14	-0.115	-0.05	-0.2
1A22	7.4	A_GLN22	0.198	0.193	0.07	-0.2
1A22	7.4	A_GLN29	0.139	0.084	0.01	-0.6
1A22	7.4	A_GLN46	0.294	0.204	1.04	0.1
1A22	7.4	A_GLN68	1.611	1.621	1.81	0.6
1A22	7.4	A_GLU174	-0.092	0.113	0.85	-0.9
1A22	7.4	A_GLU186	-0.25	-0.05	0	0
1A22	7.4	A_GLU56	1.388	1.363	0.97	0.4
1A22	7.4	A_GLU65	-0.573	-0.538	-0.11	-0.5
1A22	7.4	A_HIS18	0.927	0.252	1.84	-0.5
1A22	7.4	A_HIS21	1.353	2.638	0.78	0.2
1A22	7.4	A_ILE179	2.071	2.036	1	0.8
1A22	7.4	A_LEU45	1.687	1.607	1.15	1.2
1A22	7.4	A_LYS168	2.356	1.331	2.1	-0.2
1A22	7.4	A_LYS172	1.632	1.397	0.74	2
1A22	7.4	A_MET14	0.269	0.279	0	0.1
1A22	7.4	A_PHE176	0.838	0.838	0.6	1.9
1A22	7.4	A_PHE25	1.791	1.736	1.3	-0.4
1A22	7.4	A_SER51	0.182	0.242	-0.02	0.3
1A22	7.4	A_SER62	-0.557	-0.547	-0.06	0.1
1A22	7.4	A_THR175	0.591	0.716	2.04	2
1A22	7.4	A_TYR164	1.83	0.94	0.96	0.3
1A22	7.4	A_TYR42	2.086	1.861	2	0.2
1A22	7.4	B_ARG243	2.721	2.856	6.54	2.12

1A22	7.4	B_ARG270	0.915	0.625	-0.06	0.69
1A22	7.4	B_ARG271	3.274	2.389	1.31	0.54
1A22	7.4	B_ARG417	0.895	-0.275	0.23	0.28
1A22	7.4	B_ASN272	0.056	-0.009	0	0.28
1A22	7.4	B_ASN418	1.146	0.696	0.79	0.3
1A22	7.4	B_ASP326	-0.358	-0.118	-0.31	0.99
1A22	7.4	B_ASP364	1.081	1.801	1.37	1.49
1A22	7.4	B_GLN274	0.137	0.012	0.03	0
1A22	7.4	B_GLN366	-0.081	-0.156	0.68	0.02
1A22	7.4	B_GLN416	0.337	0.352	-0.03	0.89
1A22	7.4	B_GLU242	-0.243	0.137	0	0.18
1A22	7.4	B_GLU244	0.016	0.311	0.49	1.69
1A22	7.4	B_GLU275	-0.502	-0.382	0.09	-0.1
1A22	7.4	B_GLU320	-0.429	-0.234	0.86	-0.19
1A22	7.4	B_GLU327	1.021	0.676	1.11	0.97
1A22	7.4	B_ILE303	0.701	0.726	0.31	1.61
1A22	7.4	B_ILE305	0.51	0.445	0.13	1.94
1A22	7.4	B_ILE365	0.303	0.388	0.09	2.13
1A22	7.4	B_LYS321	0.455	-0.02	0.05	0.08
1A22	7.4	B_LYS367	0.01	-0.67	0.22	-0.02
1A22	7.4	B_LYS415	0.695	0.015	-0.02	0.79
1A22	7.4	B_SER298	-0.231	-0.251	0.29	-0.05
1A22	7.4	B_SER302	0.105	-0.015	-0.11	-0.2
1A22	7.4	B_SER324	0.069	-0.001	-0.06	0.28
1A22	7.4	B_SER419	-0.003	-0.048	0.98	0.03
1A22	7.4	B_THR273	0.007	-0.018	0	0.11
1A22	7.4	B_THR277	-0.055	-0.06	0	0.2
1A22	7.4	B_THR301	0.07	0.075	0.02	1.76
1A22	7.4	B_THR394	-0.002	-0.047	0	0.2
1A22	7.4	B_THR395	-0.077	-0.077	0	-0.09
1A22	7.4	B_TRP276	2.725	2.685	2.86	0.51
1A22	7.4	B_TRP280	0.109	0.114	0.04	-0.02
1A22	7.4	B_TRP304	5.327	5.117	5.38	4.5
1A22	7.4	B_TRP369	4.386	4.411	3.63	4.5
1A22	7.4	B_VAL371	0.53	0.52	0.26	-0.64
1A4Y	6	A_TRP261	1.209	0.689	1.05	0.1
1A4Y	6	A_TRP263	2.466	1.916	2.27	1.2
1A4Y	6	A_GLU287	0.237	-1.263	-0.02	0.1
1A4Y	6	A_SER289	-0.176	-0.421	0.55	0
1A4Y	6	A_TRP318	2.492	2.142	2.19	1.5
1A4Y	6	A_LYS320	-0.22	-0.67	-0.21	-0.3

1A4Y	6	A_GLU344	0.58	-0.69	1.37	0.2
1A4Y	6	A_TRP375	2.258	2.023	2.83	1
1A4Y	6	A_GLU401	0.472	-0.753	0.02	0.9
1A4Y	6	A_TYR434	2.838	2.278	3.03	3.3
1A4Y	6	A_ASP435	2.257	1.817	0.58	3.5
1A4Y	6	A_TYR437	2.429	1.144	3.13	0.8
1A4Y	6	A_ARG457	-0.642	-0.627	-0.04	-0.2
1A4Y	6	A_ILE459	0.356	0.011	0.63	0.7
1A4Y	6	B_ARG5	5.737	2.772	2.54	2.3
1A4Y	6	B_HIS8	0.226	0.301	0.84	0.9
1A4Y	6	B_GLN12	0.337	0.212	1.07	0.3
1A4Y	6	B_HIS13	-0.069	0.021	0.02	-0.3
1A4Y	6	B_ARG31	3.422	-0.463	2.77	0.2
1A4Y	6	B_ARG32	2.757	0.722	0.18	0.9
1A4Y	6	B_ARG33	2.132	-0.298	0	0.3
1A4Y	6	B_ARG66	1.463	0.053	0	0.2
1A4Y	6	B_ASN68	0.148	-0.157	0.52	0.2
1A4Y	6	B_ARG70	1.018	-0.112	1.02	-0.2
1A4Y	6	B_HIS84	0.13	0.015	1.01	0.2
1A4Y	6	B_TRP89	1.835	1.555	2.71	0.2
1A4Y	6	B_GLU108	-1.471	-2.126	1.73	-0.3
1A4Y	6	B_HIS114	1.315	1.285	1.69	0.65
1AHW	7	C_TYR156	2.573	2.563	4.6	4
1AHW	7	C_THR167	0.08	0.515	-0.19	0
1AHW	7	C_THR170	-0.01	0.27	-0.06	1
1AHW	7	C_LEU176	0.128	0.413	0.01	1
1AHW	7	C_ASP178	-0.55	-0.58	-0.08	-0.5
1AHW	7	C_THR197	0.069	0.194	-0.02	1.3
1AHW	7	C_VAL198	-0.121	0.224	-0.01	-0.3
1AHW	7	C_ASN199	-0.062	0.323	-0.01	1.1
1BXI	7	A_ASN24	-0.094	-0.014	0	0.14
1BXI	7	A_ASP26	0.623	0.368	0	0.34
1BXI	7	A_THR27	0.353	0.328	0.59	0.73
1BXI	7	A_SER28	0.115	0.14	0	0.17
1BXI	7	A_SER29	0.127	0.207	0.35	0.96
1BXI	7	A_GLU30	2.698	2.108	2.97	1.41
1BXI	7	A_GLU31	1.025	0.78	0.13	0.31
1BXI	7	A_GLU32	0.533	0.218	-0.01	0.22
1BXI	7	A_LEU33	1.197	1.347	1.02	3.42
1BXI	7	A_VAL34	0.832	0.837	0.98	2.58
1BXI	7	A_LYS35	-0.844	-0.499	0	0.19

1BXI	7	A_LEU36	-0.194	-0.219	0	0.91
1BXI	7	A_VAL37	0.776	0.836	0.5	1.66
1BXI	7	A_THR38	0.153	0.103	1.34	0.9
1BXI	7	A_GLU41	1.644	1.509	-0.08	2.08
1BXI	7	A_GLU42	0.82	0.545	-0.03	0.66
1BXI	7	A_THR44	-0.021	0.009	0	0.3
1BXI	7	A_GLU45	0.246	0.016	0	0.21
1BXI	7	A_HIS46	-0.172	-0.012	0	0.83
1BXI	7	A_SER48	-0.279	-0.204	-0.01	0.01
1BXI	7	A_SER50	0.129	0.054	5.4	2.19
1BXI	7	A_ASP51	1.532	1.342	0.82	5.92
1BXI	7	A_LEU52	0.065	0.095	0	0.6
1BXI	7	A_ILE53	0.36	0.435	0.17	0.85
1BXI	7	A_TYR54	3.296	3.511	2.86	4.83
1BXI	7	A_TYR55	3.317	3.242	3.31	4.63
1BXI	7	A_ASP60	0.52	0.255	0	0.51
1BXI	7	A_SER63	0.526	0.796	0	0.87
1BXI	7	A_VAL68	-0.168	-0.038	0	1.86
1BXI	7	A_ASN69	-0.032	0.028	0	0.28
1CBW	8.4	D_THR11	-0.175	-0.08	0.18	0.2
1CBW	8.4	D_LYS15	2.581	2.806	1.58	2
1CBW	8.4	D_ARG17	3.232	3.497	1.53	0.5
1CBW	8.4	D_ILE19	0.384	0.509	0.68	0.1
1CBW	8.4	D_ARG20	-0.006	0.344	-0.02	0.3
1CBW	8.4	D_VAL34	0.612	0.617	0.3	0
1CBW	8.4	D_ARG39	1.037	1.112	1.58	0.2
1CBW	8.4	D_LYS46	0.039	0.184	0	0.1
1DFJ	6	I_GLU326	1.707	2.382	1.22	1
1DFJ	6	I_TRP381	1.55	1.605	1.57	1.3
1DFJ	6	I_TRP383	2.7	2.845	3.1	2.2
1DFJ	6	I_GLU407	0.467	0.552	-0.1	1.3
1DFJ	6	I_SER409	-0.046	0.024	-0.01	0.8
1DFJ	6	I_TRP438	1.825	2.33	1.27	1
1DFJ	6	I_LYS440	-0.35	-0.585	0.38	1.3
1DFJ	6	I_GLU464	0.895	1.3	-0.12	1.6
1DFJ	6	I_GLU521	1.447	1.507	0.42	1.3
1DFJ	6	I_ARG577	-0.306	0.364	0.21	0.8
1DFJ	6	I_ILE579	1.346	1.896	0.5	0.3
1DFJ	6	I_TYR554	3.423	3.588	3.22	5.9
1DFJ	6	I_ASP555	1.422	1.447	0.16	3.6
1DFJ	6	I_TYR557	3.373	2.653	3.73	2.6

1DN2	7	A_ASN434	2.379	2.234	1.95	1.5
1DN2	7	A_HIS435	1.352	1.377	1.07	1.5
1DN2	7	A_TYR436	2.942	2.577	4.32	1.5
1DN2	7	E_VAL10	2.072	2.112	1.85	2
1DN2	7	E_TRP11	3.909	3.754	3.99	2
1DAN	7.4	T_LYS15	0.59	0.155	-0.02	-0.4
1DAN	7.4	T_THR17	-0.001	-0.081	0.13	0.1
1DAN	7.4	T_ASN18	0.348	0.393	0.04	0.2
1DAN	7.4	T_LYS20	4.846	4.491	1.5	2.6
1DAN	7.4	T_THR21	0.056	-0.204	0	-0.2
1DAN	7.4	T_ILE22	1.659	1.609	0.65	0.7
1DAN	7.4	T_GLU24	-0.281	0.184	0.64	0.7
1DAN	7.4	T_GLU26	-0.431	0.134	0	0.1
1DAN	7.4	T_LYS28	0.561	-0.269	0	0.1
1DAN	7.4	T_GLN37	1.79	1.6	1.41	0.55
1DAN	7.4	T_LYS41	0.658	-0.127	-0.04	0.35
1DAN	7.4	T_SER42	-0.175	0.015	-0.05	-0.1
1DAN	7.4	T_ASP44	0.222	0.582	0.89	0.7
1DAN	7.4	T_LYS46	0.818	-0.252	0.1	0.25
1DAN	7.4	T_SER47	-0.126	-0.016	0.56	0.05
1DAN	7.4	T_LYS48	2.121	0.841	0.43	0.4
1DAN	7.4	T_PHE50	2.54	2.17	2.61	0.4
1DAN	7.4	T_THR52	-0.05	0.075	0	0.4
1DAN	7.4	T_ASP58	0.224	0.659	1.24	2.18
1DAN	7.4	T_LYS68	0.335	-0.4	0	-0.1
1DAN	7.4	U_TYR94	2.731	1.706	2.7	1
1DAN	7.4	U_GLU99	-0.486	-0.071	0	-0.2
1DAN	7.4	U_LYS122	0.312	0.132	0	-0.1
1DAN	7.4	U_GLU128	-0.681	-0.251	-0.11	0.1
1DAN	7.4	U_ASP129	-0.585	0.015	0	0
1DAN	7.4	U_LEU133	1.413	1.408	1.62	0
1DAN	7.4	U_ARG135	2.235	1.705	0.94	0.55
1DAN	7.4	U_THR139	0.272	0.137	0	0
1DAN	7.4	U_PHE140	1.923	1.923	1.54	1.5
1DAN	7.4	U_ARG144	0.417	-0.038	0	0
1DAN	7.4	U_ASP145	-0.59	-0.08	0	0
1DAN	7.4	U_ILE152	0.031	0.181	0	0.2
1DAN	7.4	U_SER163	0.031	0.166	0.42	0
1DAN	7.4	U_THR167	-0.152	-0.292	0	0.2
1DAN	7.4	U_LYS169	0.334	-0.231	0	0.1
1DAN	7.4	U_THR172	0.003	-0.067	0	0

1DAN	7.4	U_LEU176	-0.139	-0.094	0	0.1
1DAN	7.4	U_LYS181	0.524	0.129	0	0
1DAN	7.4	U_TYR185	0.244	0.354	0	-0.35
1DAN	7.4	U_SER195	-0.065	-0.03	0	0
1DAN	7.4	U_THR203	0.095	0.26	0.21	0.1
1DAN	7.4	U_VAL207	1.261	1.391	1.12	-0.2
1DAN	7.4	U_GLU208	-0.757	-0.432	0.28	0
1F47	7.4	A_ASP2	-0.151	0.009	-0.15	0.7
1F47	7.4	A_TYR3	1.812	1.222	1.65	0.9
1F47	7.4	A_LEU4	2.038	2.023	1.94	0.9
1F47	7.4	A_ASP5	-0.333	-0.028	-0.08	1.8
1F47	7.4	A_ILE6	2.073	2.043	2.07	2.5
1F47	7.4	A_PHE9	2.332	2.272	2.43	2.5
1F47	7.4	A_LEU10	1.356	1.316	1.13	2.3
1F47	7.4	A_LYS12	0.386	-0.299	-0.12	0
1F47	7.4	A_GLN13	-0.232	-0.352	0.03	0
1FC2	7.4	C_ASN147	0.364	0.309	0.61	0.6
1FC2	7.4	C_ILE150	1.078	1.078	0.78	2.2
1FC2	7.4	C_LYS154	-0.391	-0.421	0.16	1.2
1FCC	6	C_THR25	-0.072	-0.202	0.1	0.24
1FCC	6	C_GLU27	4.008	3.993	3.21	4.9
1FCC	6	C_LYS28	2.416	2.456	0.99	1.3
1FCC	6	C_LYS31	1.901	1.661	1.91	3.5
1FCC	6	C_ASN35	2.059	2.114	1.18	2.4
1FCC	6	C_ASP40	0.562	0.082	-0.15	0.3
1FCC	6	C_GLU42	0.399	-0.001	0.03	0.4
1FCC	6	C_TRP43	2.434	2.389	2.71	3.8
1GC1	7.4	C_LYS1	-0.215	-0.075	0	0.06
1GC1	7.4	C_LYS2	-0.15	-0.185	0	-0.02
1GC1	7.4	C_LYS8	-0.402	-0.242	0	0.1
1GC1	7.4	C_ASP10	0.263	0.283	0	0
1GC1	7.4	C_THR11	-0.037	0.053	0	0
1GC1	7.4	C_THR15	-0.149	-0.044	0	0.32
1GC1	7.4	C_SER19	-0.086	-0.071	0	0
1GC1	7.4	C_GLN20	0.759	0.579	0	-0.02
1GC1	7.4	C_LYS21	-0.507	-0.192	0	-0.13
1GC1	7.4	C_LYS22	-0.322	-0.412	0	0.24
1GC1	7.4	C_SER23	0.783	0.593	0.27	0.29
1GC1	7.4	C_GLN25	0.695	0.74	0.42	0.03
1GC1	7.4	C_HIS27	0.494	0.664	0.8	0.28
1GC1	7.4	C_LYS29	2.071	2.706	2.46	0.59

1GC1	7.4	C_ASN30	-0.068	0.042	0	0.17
1GC1	7.4	C_SER31	-0.174	0.206	0	0.1
1GC1	7.4	C_ASN32	-0.208	-0.123	0	0.18
1GC1	7.4	C_GLN33	0.203	-0.247	0.03	0.1
1GC1	7.4	C_LYS35	0.802	0.747	0.56	0.32
1GC1	7.4	C_ASN39	0.079	-0.026	0	0.46
1GC1	7.4	C_GLN40	1.621	1.056	0.75	-0.41
1GC1	7.4	C_SER42	-0.244	-0.354	0.2	0
1GC1	7.4	C_LEU44	1.191	1.146	0.07	1.04
1GC1	7.4	C_THR45	0.458	0.638	0.32	-0.15
1GC1	7.4	C_SER49	0.666	0.496	0	0.6
1GC1	7.4	C_LYS50	-0.394	0.061	0	0.05
1GC1	7.4	C_ASN52	0.756	0.776	1.01	0.7
1GC1	7.4	C_ASP53	0.401	0.236	0	0.3
1GC1	7.4	C_ASP56	0.421	0.126	-0.03	-0.07
1GC1	7.4	C_ARG58	0.38	0.32	0.02	0.13
1GC1	7.4	C_ARG59	1.807	1.607	1.02	1.16
1GC1	7.4	C_SER60	-0.468	-0.538	0.13	-0.09
1GC1	7.4	C_ASP63	0.195	-0.29	-0.05	-0.32
1GC1	7.4	C_GLN64	0.992	0.772	1.16	0.44
1GC1	7.4	C_ASN66	0.069	0.084	0	-0.03
1GC1	7.4	C_LYS72	0.292	0.537	0	-0.02
1GC1	7.4	C_ASN73	0.616	0.506	0	-0.11
1GC1	7.4	C_LYS75	-0.269	-0.089	0	0.16
1GC1	7.4	C_GLU77	0.134	0.059	0	0.56
1GC1	7.4	C_THR81	0.673	0.438	0	1.5
1GC1	7.4	C_GLU85	0.372	0.047	2.1	1.31
1GC1	7.4	C_VAL86	0.103	-0.092	0	-0.07
1GC1	7.4	C_GLU87	0.587	0.167	-0.06	0.22
1GC1	7.4	C_ASP88	1.045	0.855	0	-0.07
1GC1	7.4	C_GLN89	0.005	-0.09	0	0.17
1GC1	7.4	C_LYS90	-0.476	0.079	-0.04	0.05
1GC1	7.4	C_GLU91	0.105	0.05	0	-0.13
1GC1	7.4	C_GLU92	0.079	-0.096	0	0.02
1GC1	7.4	C_GLN94	0.641	0.516	0	-0.11
1JCK	7.4	B_THR20	1.071	0.946	1.26	1.4
1JCK	7.4	B_ASN23	1.432	1.057	1.98	2.5
1JCK	7.4	B_TYR26	0.954	0.499	0.92	1.7
1JCK	7.4	B_ASN60	0.588	0.288	0.82	1.3
1JCK	7.4	B_TYR90	1.39	1.375	1	2.5
1JCK	7.4	B_VAL91	1.21	0.855	1.02	2.1

1JCK	7.4	B_LYS103	0.474	0.174	-0.26	0.4
1JCK	7.4	B_PHE176	0.545	0.05	0.63	1.9
1JCK	7.4	B_GLN210	0.907	1.447	1.12	2.5
1JRH	8	L_GLU27	0.307	0.417	0.67	0.54
1JRH	8	L_ASP28	-0.055	1.075	0.67	0.44
1JRH	8	L_TYR30	1.203	1.208	1.08	1.1
1JRH	8	L_TYR91	0.582	0.517	0.28	0.58
1JRH	8	L_TRP92	4.481	4.201	3.01	2.8
1JRH	8	L_SER93	-0.457	-0.577	0.54	-0.65
1JRH	8	L_THR94	0.469	0.444	0.36	0.38
1JRH	8	L_TRP96	1.098	0.783	0.59	1.7
1JRH	8	H_TYR32	1.023	0.643	1.63	1.4
1JRH	8	H_TRP52	2.625	2.38	1.55	2.7
1JRH	8	H_TRP53	0.738	0.638	0.73	2.4
1JRH	8	H_ASP54	0.916	1.341	1.64	1.9
1JRH	8	H_ASP55	-0.286	0.084	0	1.7
1JRH	8	H_ASP56	0.369	0.694	0.49	1.8
1JRH	8	H_TYR58	2.542	2.227	2.19	1.2
1JRH	8	H_ARG95	1.849	1.409	1.49	0.54
1JRH	8	H_PHE98	0.057	0.277	0	0
1JRH	8	H_TYR99	1.756	1.246	1.98	1.1
1JRH	8	H_HIS100B	2.717	2.722	3.38	1.7
1JRH	8	I_LYS47	2.395	2.385	1.4	3.6
1JRH	8	I_ASN48	0.134	0.059	-0.01	-0.3
1JRH	8	I_TYR49	4.207	2.872	3.87	3.4
1JRH	8	I_VAL51	1.087	1.007	1	1.9
1JRH	8	I_LYS52	2.608	2.248	2.36	3
1JRH	8	I_ASN53	2.845	2.475	2.74	3.9
1JRH	8	I_SER54	-0.045	0.105	-0.03	0.3
1JRH	8	I_GLU55	0.789	0.109	-0.1	-0.4
1JRH	8	I_ASN79	0.199	0.034	0.03	-0.4
1JRH	8	I_TRP82	1.626	1.566	1.35	4.5
1JRH	8	I_ARG84	0.654	0.539	0.37	-0.3
1JRH	8	I_LYS98	-0.354	-0.199	0.04	0
1NMB	7.4	H_TYR99	1.747	1.392	1.27	1.5
1VFB	7.2	A_HIS30	0.037	0.317	0.26	0.8
1VFB	7.2	A_TYR32	2.62	2.44	1.3	1.3
1VFB	7.2	A_TYR49	1.265	0.945	0.42	0.8
1VFB	7.2	A_TYR50	1.138	0.968	1.54	0.4
1VFB	7.2	A_THR53	0.19	0.185	0.58	-0.23
1VFB	7.2	A_TRP92	2.411	2.031	2.06	1.71

1VFB	7.2	A_SER93	-0.377	0.603	0	0.11
1VFB	7.2	B_TYR32	0.515	0.09	0.64	0.5
1VFB	7.2	B_TRP52	2.409	3.049	1.75	1.23
1VFB	7.2	B_ASN56	0.033	0.048	0.01	0.2
1VFB	7.2	B_ASP58	0.629	0.089	-0.05	-0.2
1VFB	7.2	B_GLU98	0.634	1.084	-0.03	1.1
1VFB	7.2	B_ARG99	-0.692	-1.102	0.73	0.47
1VFB	7.2	B_ASP100	1.213	0.588	3.03	3.1
1VFB	7.2	B_TYR101	3.427	3.072	3.29	4
1VFB	7.2	C_ASP18	0.749	-0.321	0.55	0.3
1VFB	7.2	C_ASN19	0.797	0.702	0.96	0.3
1VFB	7.2	C_TYR23	0.425	0.15	0	0.4
1VFB	7.2	C_SER24	-0.945	-1.375	0.97	0.8
1VFB	7.2	C_LYS116	-0.141	-0.371	0.83	0.7
1VFB	7.2	C_THR118	-0.155	-0.195	0.1	0.8
1VFB	7.2	C_ASP119	1.178	0.243	1.75	1
1VFB	7.2	C_VAL120	0.563	0.433	0.23	0.9
1VFB	7.2	C_GLN121	2.803	2.348	4.2	2.9
1VFB	7.2	C_ILE124	0.875	0.815	0.46	1.2
1VFB	7.2	C_ARG125	0.649	2.124	2.22	1.8
1VFB	7.2	C_LEU129	0.269	0.249	0.06	0.2
1VFB	7.2	B_THR30	0.055	0.05	1.22	0.09
2PTC	8.3	I_LYS15	7.017	8.747	4.16	10
1C08	7	A_ASN31	1.985	2.3	1.86	5.25
1C08	7	A_ASN32	2.379	2.749	1.27	5.2
1C08	7	A_TYR50	2.675	1.9	1.42	4.6
1C08	7	A_GLN53	0.623	0.608	0.83	1
1C08	7	A_TYR96	1.2	-0.86	0.55	2.8
1C08	7	B_SER31	-0.059	-0.064	0.45	0.2
1C08	7	B_ASP32	1.675	0.5	1.1	2
1C08	7	B_TYR33	4.298	3.863	2.9	6
1C08	7	B_TYR50	3.352	1.747	2.92	7.5
1C08	7	B_TYR53	2.635	2.36	2.04	3.29
1C08	7	B_TYR58	2.08	1.145	1.77	1.7
1C08	7	B_TRP98	3.193	2.813	1	5.5
1C08	7	C_ASP101	1.634	2.024	1.02	1.5
1C08	7	C_HIS15	0.304	0.259	0.09	-0.5
1C08	7	C_TYR20	3.372	2.422	2.72	5
1C08	7	C_ARG21	4.168	1.213	3.47	1
1C08	7	C_TRP63	1.622	1.362	0.83	0.3
1C08	7	C_ARG73	0.616	0.181	0.62	-0.2

1C08	7	C_LEU75	2.226	2.131	1.3	1.25
1C08	7	C_THR89	0.1	0.09	0.17	0
1C08	7	C_ASN93	1.048	1.003	1.53	0.6
1C08	7	C_LYS96	3.274	3.589	2.13	7
1C08	7	C_LYS97	4.182	2.977	1.42	6
1C08	7	C_ILE98	0.143	0.113	0.1	-0.1
1C08	7	C_SER100	0.054	-0.196	0.95	0.25
1C08	7	C_ASP101	1.634	2.024	1.02	1.5
1BRS	8	A_LYS27	2.968	2.773	1.91	5.4
1BRS	8	A_ASP54	-0.058	-0.148	-0.04	-0.8
1BRS	8	A_ASN58	-0.04	-0.41	-0.03	3.1
1BRS	8	A_ARG59	5.054	5.024	3.01	5.2
1BRS	8	A_GLU60	0.021	-0.509	1.65	-0.2
1BRS	8	A_GLU73	-0.787	-0.972	-0.2	2.8
1BRS	8	A_ARG87	3.543	2.733	4.44	5.5
1BRS	8	A_HIS102	3.377	3.852	5.08	6
1BRS	8	D_TYR29	3.423	2.553	3.13	3.4
1BRS	8	D_ASP39	2.727	3.032	9.4	7.7
1BRS	8	D_ASP35	2.557	2.102	1.42	4.5
1BRS	8	D_THR42	0.578	0.823	1.69	1.8
1BRS	8	D_GLU76	0.996	0.876	1.54	1.3
1BRS	8	D_GLU80	0.594	0.014	-0.12	0.5
		**RMSD:**	**1.15**	**1.12**	**1.31**	
